# Broad Tuning of Paper Microfluidic Properties by Covalent Surface Modification for Precise Flow Control and Sensing

**DOI:** 10.1021/acsabm.4c01812

**Published:** 2025-04-17

**Authors:** Canan Aksoy, Ischa van Kesteren, Han Zuilhof, Gert IJ Salentijn

**Affiliations:** † Laboratory of Organic Chemistry, Wageningen University, Helix Building 124, Stippeneng 4, Wageningen 6708 WE, the Netherlands; ‡ Wageningen Food Safety Research, Wageningen University & Research, Wageningen 6700 AE, the Netherlands; § College of Biological and Chemical Engineering, Jiaxing University, Jiaxing 314001, China

**Keywords:** paper microfluidics, microfluidic paper-based analytical devices, paper-based sensing, on-site analytical devices, paper surface modification, covalent functionalization

## Abstract

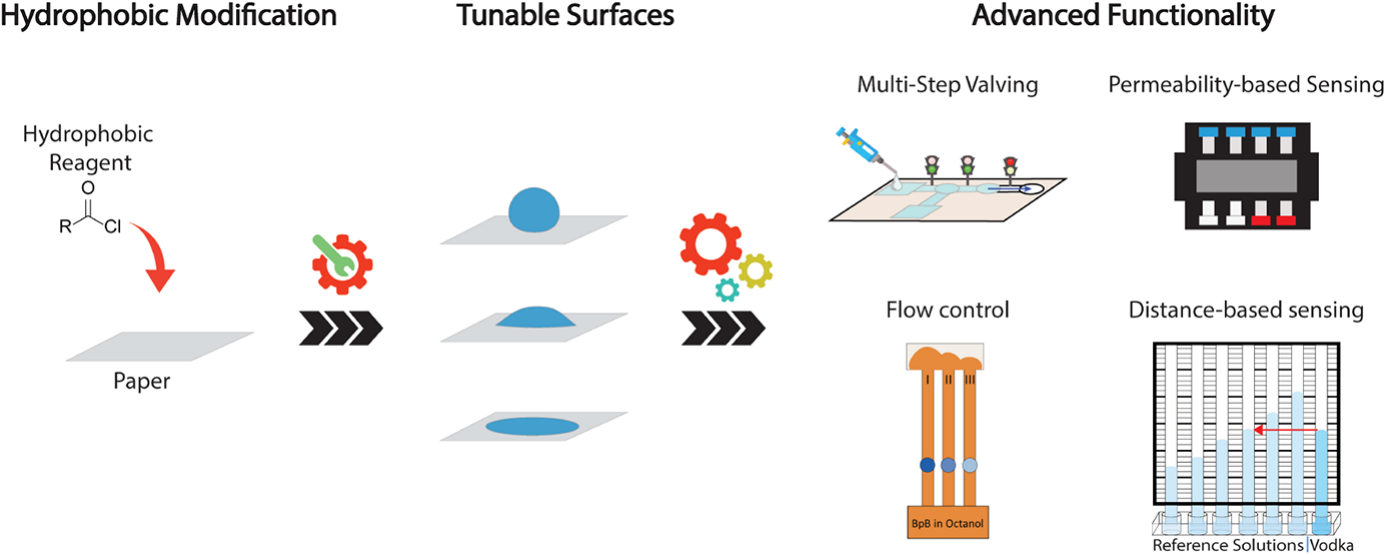

In an effort to innovate on-site sensing platforms for a wide range of analytes in different matrices, microfluidic paper-based devices (μPADs) are promising candidates to bring the lab to the sample, as they allow passive, capillary-action-driven flow. Their use, however, is somewhat limited by the fact that the integration of advanced functionality and flow control is difficult. Although recent progress in this area has led to the development of on/off-valving and timing control of flow by changing the chemical and physical properties of paper, precise control over flow in paper microfluidics remains challenging. Here, we propose the use of a simple covalent modification of cellulose paper to tune its surface properties, thereby introducing a broad range of functionality and applicability. For this purpose, fatty acyl chlorides with different chain lengths were used as hydrophobic reagents to change the surface properties. The modified paper was characterized by FTIR-ATR, static water contact angle measurements, and capillary flow properties (permeability, maximum flow distance, and flow rate). The produced papers were then applied in several proof-of-concept devices to demonstrate their potential in sensing and actuating for improved on-site analysis. We demonstrate how precisely modified paper can be used for surface tension measurements and multistep valving based on its wickability for solutions of varying surface tensions, for the determination of ethanol concentration in water by monitoring the maximum flow distance in a 3D-printed device, and for the optimization of on-paper liquid–liquid extraction via fine-tuned control of capillary flow rates.

## 1 Introduction

Point-of-care (PoC) and point-of-need (PoN) analyses are experiencing rapid growth, driven by their successful implementation as early warning tools in medical diagnostics, and in maintaining food and environmental safety.^1^ A recent success story is, of course, the global application of rapid tests to diagnose SARS-CoV-2 infections, underscoring the increasing importance of timely diagnostics to limit the spread of diseases. Key to the success of such tests is that they are affordable, sensitive, specific, user-friendly, rapid and robust, equipment-free, and deliverable to the end user (ASSURED).^2^ This stands in stark contrast to more conventional, lab-based methods, which – despite their superior analytical performance in the identification and quantification of analytes – are time-consuming, requiring complex procedures, sophisticated laboratories with expensive equipment, and professionals trained in analytical science.^3^ It is now widely accepted that fast, on-site methods can overcome many limitations of lab-based methods and complement them by decentralized screening approaches.^4−6^

Prime examples of on-site analytical devices can be found in the field of microfluidics.^7^ Microfluidic devices are miniaturized, often chip-based systems designed to precisely manipulate fluid flows by changing the fluid and/or material properties or device parameters in a well-controlled fashion. Such analytical devices have many advantages, such as requiring only small amounts of sample, integrating different functional elements, and offering high cost efficiency.^7−9^ A special subcategory of microfluidics is represented by microfluidic paper-based analytical devices (μPADs), which are especially promising for on-site analytical applications, given the fact that paper is inexpensive, flexible, lightweight, and compatible with many solvents.^10,11^ As paper is fabricated from hydrophilic cellulosic fibers and has a tortuous, porous structure,^10,12^ it enables passive, capillary-driven flow, thereby removing the requirement for an external pump to generate flow.^11,13,14^ As a result, the material can be used as a substrate in the development and production of many different quick tests, which are typically referred to as paper microfluidics.

In paper microfluidics, the passive capillary flow can be described by the Washburn equation, which characterizes the flow in porous media by assuming that it can be treated as a bundle of parallel capillary tubes with a uniform radius. It is simplified to the Lucas-Washburn equation eq 1 for horizontal flow by eliminating the gravitational force from the equation.^15,16^ This model can be used for vertical flow when the hydrostatic pressure is negligible compared to the capillary pressure. Thus, the flow is primarily governed by the balance between surface tension and viscous forces.^17^ In this equation, *l* is the distance traveled by the solution, *t* is the flow time, γ is the surface tension of the liquid in air–liquid interface, ϕ is the contact angle between the liquid and the solid surface, *r* is the radius of the capillary tubes, and μ is the dynamic viscosity of the liquid.^15,16^
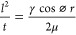
1

Capillary flow in paper can be directed through confined hydrophilic regions, typically referred to as “channels”, via hydrophobic patterning of impermeable “walls”, e.g., by wax printing or PDMS coating.^18−20^ In addition to confined flow regions, sample and detection zones can also be created to obtain passive flow from one region to another for detection and quantification of analytes.^18,21,22^ The advantage of passive flow in paper, however, also represents its major limitation, namely, that paper-based devices lack any control over flow rates, since the flow occurs spontaneously. This drawback is one of the major challenges in developing fully automated, robust μPADs as sensitive analytical tools.^23^ Precise flow control in paper microfluidics is therefore highly needed to allow the integration of functional operational elements in such devices, e.g., for sample handling, sequential analyte transfer, and ultimately to improve performance and applicability.^23−25^ Hence, a significant amount of scientific work has been devoted to finding ways to manipulate flows in paper microfluidics and extend its scope.^26^

Following different strategies, some degree of tunability in the microfluidic properties of paper has been achieved, including geometrical and chemical modifications. Geometrical alteration was used as a flow manipulation strategy by Fu et al., who demonstrated that having a wider central segment of a paper strip, while maintaining identical sizes for the segment that was in contact with the solution, resulted in an increased travel time for the same linear distance.^27^ Park et al. created a pressed region on paper, which led to a delay in the arrival time of the sample coming from it, due to a reduced internal volume and pore size, so that they could transfer multiple analytes in a sequential manner to develop a multistep dipstick.^28^ Liu et al. applied laser carving within patterned paper-based channels to obtain microcracks. These microcracks are essentially miniature channels, through which fast capillary flow can be obtained, leading to a significant increase in the overall flow rate.^29^

A second and broader strategy to tune the microfluidic properties of paper is to change its (physico-)chemical properties, thereby affecting the interaction between cellulose and liquid, and, as a result, capillary flow.^26^ Several studies have so far focused on two main categories: (i) physical deposition of hydrophobic materials, such as wax, to decrease or block its pores and alter porosity and surface energy and (ii) covalent, chemical modification of paper, which mainly influences the surface properties of cellulose fibers, rather than the pores. In the first category, Strong et al. achieved tunable time delay by varying the amount of wax loaded on paper,^30^ and Chen et al. have designed a wax-printed valve that separated the sample and detection regions, so that it was “off’ in aqueous media, while it could be turned “on” by wetting it with a drop of organic solvent to achieve a controlled mixing, incubation, and flow.^31^ While those methods based on physical deposition of, for example, wax are easily incorporated, such coatings are affected by solvents and will actually partially dissolve, thereby changing the device – and thus its detection properties – over time and contaminating the sample with the hydrophobic material. In contrast, when using covalent attachment, it has been demonstrated that functional elements can actually withstand multiple wetting and drying processes and thus be restored to their starting state. For example, Salentijn et al. applied covalent hydrophobic patterning of paper using alkyl ketene dimer to achieve solvent-dependent on/off valving with hydrophobic regions that were selectively permeable to solvents.^32^ Analogously, Li et al. systematically varied the wettability of paper (hydrophilic, hydrophobic, oleophilic, and oleophobic) by applying fluoro-silanization, followed by oxygen plasma etching to adjust the surface properties.^33^ It was demonstrated that all combinations of these surface conditions could be obtained with the suggested two-step method, so that modified papers could be used in several applications such as oil–water separation and measurement of surface tension. While Li et al. worked with extreme conditions to achieve distinct surface properties with plasma treatment, Rosso et al. focused on finer tuning. It was demonstrated how plasma treatment gently modifies surface properties, particularly on silicon and silicon nitride surfaces. This work underscored the potential of the technique for broader applications requiring precise surface adjustments, which could potentially be adapted for paper modification as well.^34^

Despite these advances, there is currently a lack of an easily accessible and adaptable toolbox to functionalize cellulose paper in a broad range, allowing precise tuning of its flow properties for application in paper microfluidics. For example, in those previous studies, mostly binary (wicking versus nonwicking) systems were developed, or systems with a single type of chemical modification were studied to observe the effect on the capillary flow rate. However, gradually altering the surface energy of paper with various covalent modifications has not been studied, while this would, in principle, yield many levels of controlled wettability that can respond sensitively with respect to small changes in the surface tension of the solvent.

In this work, we therefore set out to study the influence of small variations in covalently attached hydrophobic reagents (fatty acyl chlorides of different lengths) on the microfluidic properties of paper. A broad range of covalent surface modifications were performed to achieve precise control over the wettability and “wickability” (or permeability) of papers, as well as over the flow properties of solutions with varying surface tensions through those papers. We optimized the reaction conditions so as to allow optimal sample analysis, following correlations resulting from eq 1. The characteristics of the modified papers were then investigated in terms of their permeability (whether the paper wicks the solution or not), maximum flow distance (how far the solution flows before it stops), and flow rate (how fast the solution wicks through the modified paper). Finally, in order to demonstrate the applicability of the proposed toolbox for tuning the properties of paper microfluidic devices, several paper-based applications were developed, including surface tension measurement, multistep valving, alcohol concentration determination, and optimization of paper-based liquid–liquid extraction.

## 2 Experimental Section

### 2.1 Materials

Whatman chromatography (CHR) paper grades 1, 3MM, and 17 were used for the paper; these three paper types vary predominantly in their thickness and water flow rate (see Table S1). 1-Butyryl chloride, C4 (98%), 1-hexanoyl chloride, C6 (97%), 1-octanoyl chloride, C8 (99%), lauroyl chloride, C12 (98%), palmitoyl chloride, C16 (98%), molecular sieves 4 Å (beads 8–12 mesh), and 1-octanol (≥99%) were purchased from Sigma-Aldrich. Pyridine (>99.0%) and bromophenol blue (ACS, crystalline) were supplied by Alfa Aesar. Sulfanilamide (98%), sodium nitrite (99% extra pure), phenoxyacetyl chloride, POAC (98%), phenylacetyl chloride, PAC (98%), and ethyl succinyl chloride, ESC (95%), were obtained from Acros Organics. *N*-(1-Naphthyl)ethylenediamine dihydrochloride (ACS, powder) was purchased from Thermo Fisher. Toluene (ACS reagent grade, ≥99.7) was supplied by Honeywell Riedel-de Haën, Seelze, Germany. Acetone (analytical grade, ≥99.8%) and dimethylformamide, DMF, were obtained from Rosny-sous-Bois and Fontenay-sous-Bois, France. Ethanol, EtOH (96.2% v/v) (Boom B.V., Meppel, The Netherlands), was used for washing paper. Absolute ethanol (Biosolve Chemie, Valkenswaard, The Netherlands) was used for the preparation of sample solutions. Milli-Q water (18.2 MΩ·cm, Milli-Q Integral 3 system, Millipore) was used for washing papers and preparing solutions. Dr. Oetker food dyes and vodka (37.5% EtOH v/v) were purchased from a local grocery store. Whatman CF1 grade sample pad (Whatman, GE Healthcare, Eindhoven, The Netherlands) and a plastic adhesive backing card (G and L, San Jose, CA, USA) were used in sensing devices. Polylactic acid, PLA, (1.75 mm diameter) black or white filament was used in 3D-printed holders and purchased from 123-3D.nl (Almere, The Netherlands).

### 2.2 3D-Printed Holders

SolidWorks 2019 was used to draw 3D-printed devices and holders. The drawn models were saved as *.STL files. These files were sliced by using Ultimaker Cura 4.9.0, where the bed and PLA filament print temperatures were set to 60 and 200 °C, respectively. The print speed was fixed at 50 mm/s. Printing resolution was set to 0.16 and 0.2 mm for the flow measurement holder and the permeability-based sensing device, respectively. Their GCode was then loaded to a Creality 3D Ender-3 Pro Printer, and the devices were printed in PLA.

### 2.3 Covalent Modification of Cellulose Paper

**Figure 1:**
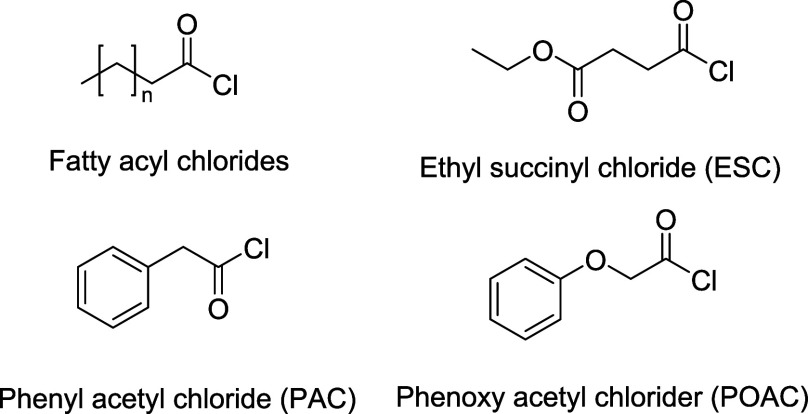
Chemical structures of the reagents used in paper modification, where *n* = 1, 3, 5, 9, or 13 for fatty acyl chlorides of C4, C6, C8, C12, and C16, respectively.

Papers were modified by an acylation reaction (Figure S1). The procedure used was adapted from Freire et al. (2006).^35^ Before use, the solvent (DMF) and pyridine were dried overnight over 4 Å molecular sieves, which were activated in a vacuum oven at 120 °C overnight beforehand. Papers were cut into 5 mm strips with a BIODOT CM5000 Guillotine Cutter. Various reagents were used for the modification (Figure 1) within a broad polarity range (see Table S2 for *n*-octanol–water partitioning coefficients), and the amounts of reagent used were adjusted according to the amount of available OH groups in the paper; this was expressed as the equivalent (equiv) of the hydrophobic reagent (acyl chlorides) to hydroxyl groups in cellulose (Table S3 and eq S1).

Papers were precut into 6.5 cm × 0.5 cm strips, and 30 strips were weighed, resulting in a total mass of 845 ± 5 mg. The required volume of the hydrophobic reagent for this amount of paper, calculated by eq S1, was placed into a 500 mL Erlenmeyer flask. Dry DMF (70 mL/g cellulose), dry pyridine (in the same equiv as acyl chloride), and paper strips were then added. The reaction was conducted at room temperature (RT), 40 °C, and 80 °C, and with various reaction times (1, 2, 4, 6, 16, and 24 h). After the reaction, the covalently modified papers were sequentially washed with toluene, acetone, EtOH, water, and EtOH and stored in EtOH overnight. Afterward, the papers were dried in a vacuum oven at 50 °C overnight. The reaction conditions were varied to optimize the modification in terms of reproducibility and to diversify the paper surface properties over a broad range (Table S4).

Modified papers are named after the reagent, the equivalent amount used in the reaction, the reaction temperature, and the reaction time, so that, e.g., C4(0.8)-80T6h refers to modified paper for which C4 acyl chloride was used in 0.8 equiv during the acylation reaction at 80 °C for 6 h.

### 2.4 Characterization of Modified Papers

After the modification of the papers, their surface chemistry, hydrophobicity, and morphology were characterized with Fourier-transform infrared (FTIR) spectroscopy, static contact angle measurements, and a scanning electron microscope (SEM), respectively.

#### 2.4.1 ATR-FTIR Spectroscopy Analysis

The chemical composition of the modified and unmodified papers was characterized using FTIR measurements (Bruker Tensor II spectrometer, platinum attenuated total reflection (ATR) accessory, 32 scans, resolution of 4 cm^–1^, spectral range of 700–4000 cm^–1^) by pressing the paper sample with a fixed clamp to increase the contact between the sample and the ATR accessory. First, a background measurement was performed with ambient air. The spectra of the sample papers were then acquired. Measurements were performed for different batches of a type of modified paper by selecting a random paper from each batch, and measurements were performed on at least three different spots for each sample. Their chemical stability was also assessed by placing water-permeable ESC(0.8)-80T6h paper in water and aqueous solutions at pH 2, 4, 10, and 12 for 1 h, while C8(1)-80T6h paper was placed in a 1:1 (v/v) mixture of EtOH and aqueous acidic and alkaline solutions of pH 2, 4, 10, and 12. The papers were then dried in a vacuum oven for 1 h, after which they were analyzed by ATR-FTIR.

#### 2.4.2 Contact Angle Measurements

The hydrophobicity of modified papers was characterized by measuring the static water contact angles (WCA), as well as the contact angles of binary mixtures of water and EtOH. Contact angles were measured using a KRÜSS Drop Shape Analyzer (DSA 30). First, paper samples were taped onto a glass slide with a piece of double-sided tape to align them horizontally. 3 μL droplets of the solutions were dispensed on the paper surface by using a syringe; then, the contact angle between the paper surface and the liquid drops was measured with an integrated camera using the sessile drop method in the software, ADVANCE. For WCA measurements, Milli-Q water was used. Aqueous EtOH solutions in varying concentrations were applied to observe changing contact angles with varying EtOH content and to determine the EtOH concentration at which the solution would fully wick into the paper. Three droplets (*n* = 3) were measured on a single paper strip.

#### 2.4.3 SEM Analysis

SEM analysis of modified and unmodified papers was conducted at room temperature using a JEOL JAMP-9500F Field Emission Auger Microprobe system. Samples were sputter-coated with gold to enhance the conductivity for imaging. The sputtering process was performed with a JEOL JFC-1300 Auto Fine Coater by applying two layers, each with a coating duration of 40 s. SEM images were acquired with a beam energy of 5 keV to optimize the resolution and minimize sample damage.

### 2.5 Characterization of Capillary Flow Properties

Several flow properties of the differently modified papers were investigated and compared with those of unmodified papers. Modification was used to control the surface energy of the paper, thus influencing the (i) wettability of papers, (ii) wicking/nonwicking of solutions with varying surface tensions, (iii) capillary flow distance, and (iv) flow rates of liquids traveling through the paper. These characteristics of the modified papers were assessed using solutions of varying surface tension, which were obtained by making binary mixtures of water and EtOH. Specifically, three types of measurements were conducted, namely, permeability-based, distance-based, and time-based analyses, by monitoring (i) if the solutions wicked into the paper or not, (ii) how far the solutions flowed in the paper, and (iii) how fast they flowed through the paper, respectively.

#### 2.5.1 Permeability-Based Analysis

Permeability-based analyses were performed to assess whether an aqueous EtOH solution would wick into the tested paper or not. To do so, droplets of water with increasing percentages of EtOH were applied to the paper to observe whether they would (partially) wick into the paper within a couple of seconds. The minimum required EtOH concentration for a solution to fully wick into a modified paper was defined as the critical wicking concentration (CWC) and was determined for each paper. A Krüss drop shape analyzer was used to record a video of the wicking of droplets of 3 μL, with the EtOH percentage increasing in 1% steps around the estimated CWC (Figure S2A). In another type of experiment, 5 μL of solution was manually deposited on the paper, and wicking was observed visually to demonstrate its practical applicability. This method also allowed us to perform quick experiments for smaller increments of EtOH concentration, changing in 0.5% steps (Figure S2B).

#### 2.5.2 Distance-Based Analysis

In wicking experiments with water/EtOH solutions of a fixed % EtOH on modified papers, the solution would typically move through the paper up to a certain distance and then stop. Distance-based analysis was performed to investigate how far a liquid sample would travel along the paper before stopping. The experiment was performed by vertically dipping strips of modified paper into the wells of a 96-well plate, each filled with 300 μL of varying % EtOH in water. Strips could be reproducibly positioned by using a 3D-printed holder (Figure S3). After the capillary flow stopped, a photograph of the device was taken with a smartphone, and the distance traveled by the liquid was measured from the image against the grid of reference lines. In the initial benchmarking experiments, the analysis was performed either (i) on an open bench, (ii) in a closed container (thin-layer chromatography, TLC, chamber) filled with ambient air, or (iii) in a chamber saturated with EtOH, and the results were compared to understand the effect of EtOH evaporation on the maximum flow distance. All subsequent experiments were performed in a TLC chamber filled with ambient air.

#### 2.5.3 Time-Based Analysis

The 3D-printed holder that was used in distance-based measurements was also utilized to monitor the linear relationship between the square of the flow distance and flow time (eq 1) through the differently modified papers. Similar to distance-based measurements, strips were placed into the solutions in the well plate, and the flow was recorded with a smartphone. The reference gridlines behind the paper holder were used to determine the distance traveled by the solution at specific times.

## 3 Results and Discussion

### 3.1 Covalent Modification

The surface properties and functionality of modified papers were studied for different reagent types, amounts, starting papers, reaction times, and temperatures (Table S4). All these parameters had an influence on not only the surface chemistry of the paper but also its physical structure. This is relevant since the combination of physical alterations and covalent surface modifications together tunes the paper microfluidics. A notable physical change was an increased thickness of the paper after the covalent modification (Figure S4), where the influence varied for different reaction conditions and paper types used. Longer fatty acid chain lengths or higher reaction temperatures, for example, resulted in thicker modified papers. In particular, thick papers were obtained when using Gr17 paper, where the thickness increased from approximately 1 to 4 mm. Although the Gr17-C8(2)-80T6h paper was impermeable to water, the highly swollen structure of this paper made it highly opaque, thereby making it impossible to follow the flow of liquid in the paper by eye. Regarding the starting materials, Gr3MM paper was modified under a single set of conditions only since it did not show a difference compared to Gr1 paper in terms of maximum flow distance in early experiments. Gr17 paper, on the other hand, allowed for further diversification of the approach. Finally, a threshold for the number of equiv of the reagents used in modification was identified for modified papers to become impermeable to water; thus, lower numbers of equiv than this threshold were not explored (Table S4).

### 3.2 Characterization

#### 3.2.1 ATR-FTIR Spectroscopy Analysis

After modification, all papers were analyzed with ATR-FTIR spectroscopy, and the spectra were compared to those of bare paper to qualitatively investigate the degree of covalent modification. All spectra were normalized with respect to the C–O–C vibration peak at 1055 cm^–1^, which was assumed to remain unchanged after modification.^36−38^

For bare paper (Figures S5 and S6), broad peaks were observed between 3600–3100 and 3000–2800 cm^–1^, which were attributed to hydroxyl (O–H) stretch and alkyl (C–H) stretch, respectively.^35,39^ For modified papers, those same bands were observed, where convoluted peaks at 2924 and 2854 cm^–1^ represented −CH_2_ symmetric and antisymmetric stretch vibrations, respectively. Importantly, a peak was observed at 1740 cm^–1^, which was attributed to an ester (—O—C=O) stretch, originating from the covalent attachment of the fatty acyl chloride to hydroxyl groups on cellulose.^35,37^ This signal was the first qualitative indicator of successful covalent modification. Moreover, no signal was obtained for the acyl halide, which should have been observed at 1803 cm^–1^ in the case of having physically adsorbed reagent residues, rather than covalent attachment. The only exception to this was seen with C16(2) papers for reaction times longer than 1 h, likely due to the excessive use of this very hydrophobic reagent (Figure S7).^40^ Additionally, it was observed that while the ratio of the alkyl stretch peak intensities over ester peak intensity increased with an increasing chain length of the fatty acyl chloride used, the ester peak intensities themselves were similar (Figure S5), suggesting a similar amount of reagents attached to the paper surface. It was also observed that esterification could be successfully achieved for shorter reaction times than 6 h: reaction times of 1, 2, and 4 h for C16(2) and C8(2) at 80 °C all resulted in covalent attachment, with similar peak intensities (Figures S7 and S8). The effect of the reaction time was then further investigated in terms of the WCA and CWC for optimization and reproducibility purposes.

ATR-FTIR spectra were acquired from three spots for a single strip of C8(1)-80T6h and C12(1)-80T6h papers and three spots for two equally treated C4(1)-80T6h and C6(1)-80T6h papers (Figure S9). The relative peak intensities of the related functional groups were highly reproducible, not only for different locations on a single strip but also for different batches of the same modifications. It was also observed that esterification could be successfully achieved with C8(1) at RT, 40 °C, and 80 °C for 6 h of reaction time, with increased esterification at higher temperatures (Figure S10). Finally, for different starting papers, the most efficient modification was observed for Gr1 paper, while the least efficient modification was observed for Gr17 paper (Figure S11). The variations in geometry, such as paper thickness, porosity, and pore size, likely account for this.

Finally, the stability of the modified papers was examined at various pH values. ATR-FTIR spectra of papers stored for 1 h under the various conditions showed no substantial changes, peak formations, or changes in peak intensities, particularly in characteristic molecular features (hydroxyl, alkyl, and ester stretching vibrations; Figure S12). This suggested that the modified papers preserved their structure in acidic (pH 2 and 4) and alkaline (pH 10 and 12) media, indicating resistance to hydrolysis or chemical degradation.

#### 3.2.2 Contact Angle Measurements

**Figure 2:**
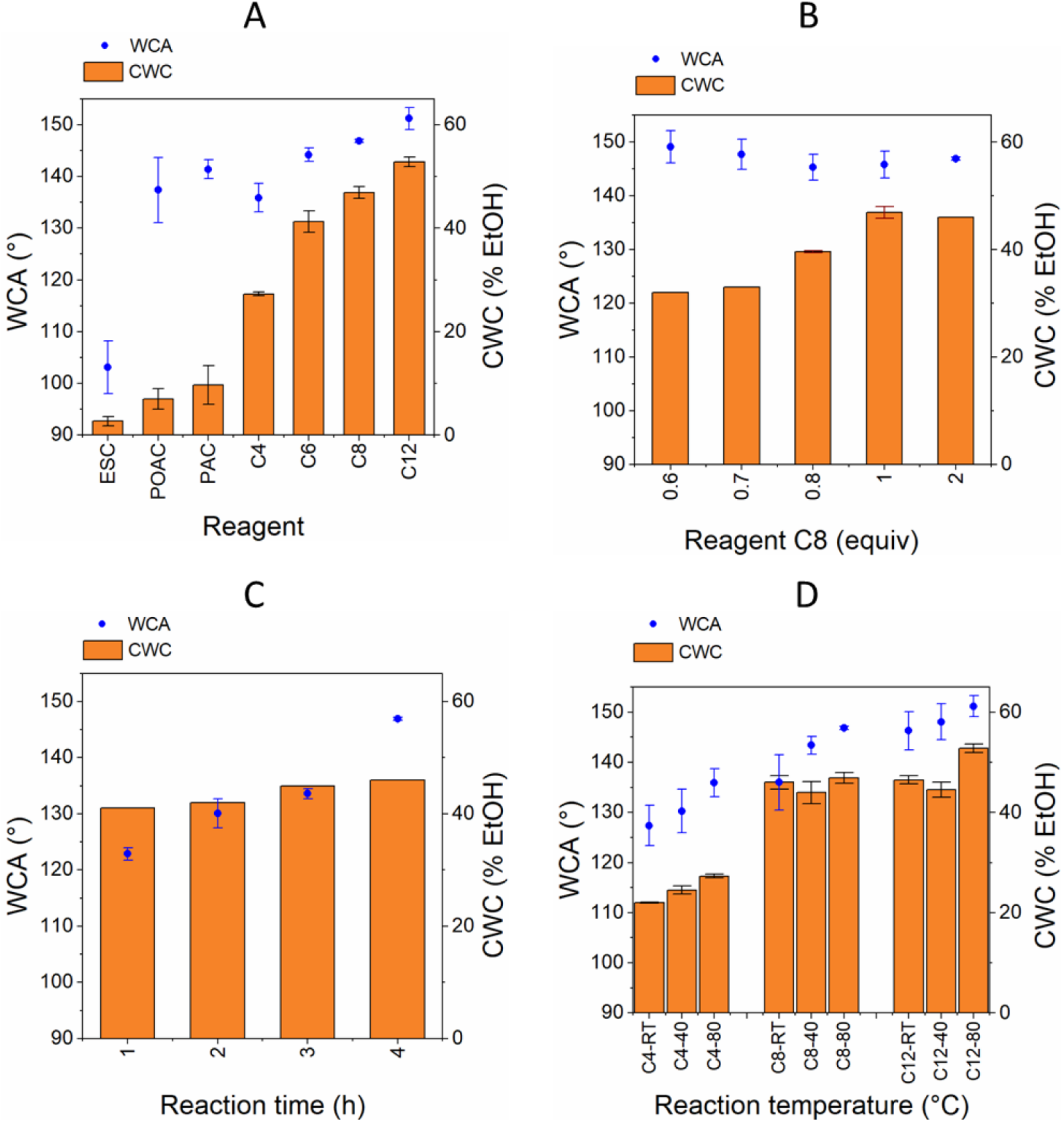
WCA and CWC of modified Gr1 papers from the reaction of (A) 1 equiv of various reagents at 80 °C for 6 h; (B) varying equiv of reagent C8 at 80 °C for 6 h; (C) varying reaction time of reagent C8 at 80 °C; and (D) varying reaction temperature of reagents C4, C6 and C8 for 6 h. Error bars represent standard deviation; see Table S5 for details.

As the aim of this work was to control paper microfluidic flow by tuning the surface wettability, contact angle measurements were first performed to provide crucial insights into the hydrophobicity of the modified papers. The manner in which the WCA was influenced by reagent type, reaction temperature, reagent amount, and the reaction time was plotted together with the CWC of those modified papers in Figure 2 (see also Table S5 for WCA and CWC of papers modified by different reagent types, amounts, reaction temperatures and times, and types of paper). The results varied in a broad range with the lowest WCA of 103° ± 5° and the highest WCA of 158° ± 3° found for ESC (1)-80T6h and C16(2)-80T6h on Gr1 paper, respectively. As expected, the WCA of modified papers increased with longer chain lengths of the fatty acyl chlorides. Similarly, WCA values increased from ESC, via POAC, to PAC, consistent with their decreasing polarity (Figure 2A). In addition, the WCA was not affected by the reagent amount over the range tested (from 0.6 to 2 equiv of acyl chloride) (Figure 2B), while it was lower for shorter reaction times (varied from 1 to 6 h, Figure 2C) and lower reaction temperatures (varied from RT to 80 °C, Figure 2D).

#### 3.2.3 SEM Analysis

To observe potential morphological differences between modified, heated, and untreated papers, SEM images were acquired for the bare grade 1 paper, DMF-treated papers at RT and 80 °C, and C16(0.6)-80T6h modified papers (Figure S13). Untreated papers exhibited a well-defined fiber network with clearly distinguishable individual fibers. In contrast, the treated papers showed a noticeable loosening of the fibers. Their shapes appeared altered and intertwined more closely. This structural alteration suggests that the treatment process with or without the presence of FA reagents affected the fiber integrity, thereby potentially influencing the papers’ physical properties and, as a result, capillary flow behavior. This result aligns with previous reports^35^ in which damage to cellulose fibers after the acylation reaction in DMF was observed.

### 3.3 Characterization of Capillary Flow Properties

#### 3.3.1 Permeability-Based Analysis

To demonstrate that the surface properties of paper can be tuned by covalent modification to control capillary flow through paper, permeability tests were performed in addition to the reported WCA measurements. While the WCA gives a clear indication of the hydrophobicity of the modified papers, permeability-based analysis provides insight into the interaction between the papers and liquids with surface tension lower than that of water. Aqueous EtOH solutions with various % EtOH were thus used to establish the wicking/nonwicking response of the modified papers. Bare (unmodified) cellulose paper is permeable to water due to the strong polar interaction between cellulose and water and its porous structure, whereas (sufficiently) modified hydrophobic papers are impermeable to water due to lowered surface energy. With modified papers, the wettability increased with higher % EtOH, due to the decreasing surface tension of the solution, until the solution wicks into the paper. The CWC was determined for varying reagent types, reagent amounts, and reaction times and temperatures (Figure 2; CWC values of all modified papers can be found in Table S5). Despite the fact that the CWC has a generally increasing trend with decreased polarity, for some modifications, the same trend could not be observed. It was expected to see higher CWC for POAC and PAC papers than for C4 papers due to their lower polarity, based on their partition coefficient (Table S2), as observed in the WCA values; however, they resulted in lower values (Figure 2A). This result may imply that it is not only the polarity of the reagent and the resulting surface chemistry but also physical changes occurring in the paper during modification that contribute to the wicking properties of the modified papers. The physical change could be observed most dramatically for Gr17 paper modified with C8(1) at 80 °C for 6 h (Figure S4), where the thickness increased by a factor of 4.

In terms of the amount of the reagent used, a clear impact on the CWC was observed (Figure 2B). For example, a stark difference was observed between C8(0.6) and C8(0.5) papers, with the former having a CWC of 32% EtOH, while the latter simply imbibed water. We defined the minimum required amount of the reagent for modification to achieve a CWC as a threshold equiv. This value was observed at 0.4 equiv for C16 and C12, 0.5 equiv for C8, C6 and C4, and 0.9 equiv for ESC, POAC and PAC. Above these thresholds, for all reagents, an increase in the number of equiv would lead to a rise in CWC within the tested range of equiv. It was observed that using 2 equiv could result in residual reagents remaining in the paper, as mentioned in the ATR-FTIR analysis of C16(2)-80T6h paper. Additionally, this amount of reagent did not produce an increase in WCA and CWC for C8 compared to 1 equiv, and only a slight increase for C12 and C16; therefore, amounts above 2 equiv were not investigated. The total range of CWC values obtained with the presented array of covalent modifications covers 9.5–57.7% EtOH in water.

The reaction time was optimized to reproducibly produce hydrophobically modified papers in the shortest time possible. For all modifications, an increase in the reaction time up to 6 h resulted in an increase of WCA and CWC (Figure 2C). Furthermore, for reaction times of 1, 2, and 4 h, it was more challenging to obtain repeatable results, but at 6 h, a robust and easily reproducible modification was established (Table S5). Extending the reaction time to 16 and 24 h did not lead to different WCA and CWC for C4 compared to 6 h, while C12 resulted in higher CWC but similar WCA compared to 6 h, likely related to a change in the structure as a result of the long exposure to solvents under elevated temperature. Therefore, a 6 h modification time was selected for further experiments.

Next, the influence of the reaction temperature was investigated. It was observed that modified papers have, in addition to their altered wetting properties, altered structures as well (Figure S4). It was therefore investigated whether modification could be carried out under milder conditions to prevent possible drawbacks of these structural changes. Reactions were carried out at RT, 40 °C, and 80 °C for C4, C8, and C12 reagents with Gr1 paper. Temperature elevation during modifications led to higher WCA and CWC for C4 and C12; for C8, even though the WCA increased with the reaction temperature, the CWC did not show a pronounced difference (Table S5). These differences might be explained by the fact that especially at an elevated reaction temperature, the structural properties of the paper also change, favoring the wicking into those papers. On the other hand, the elevated temperatures also lead to an increased hydrophobicity (as evidenced by the increasing WCA), and so these two effects influence the CWC in opposite directions.

Finally, different papers as starting materials were studied; a thicker paper with a similar flow rate to Gr1 paper (Gr3MM) and a thicker paper with a higher water flow rate (Gr17) were selected for this purpose (Table S1). The WCA and CWC results of Gr3MM paper were obviously distinct compared to Gr1 paper for the C8(1)-80T6h modification, while Gr17 paper wicked water when produced under the same modification conditions. The first important observation was the significant change in shape after modification for Gr17 paper. The reaction at 80 °C yielded thicker, sponge-like modified papers (Figure S4), whereas the modification carried out at RT resulted in a paper that, to the naked eye, looked much more like the starting material. The required equivalents of reagent to obtain Gr17 paper that would repel water were also greater than those for Gr1; this can be explained by the fact that capillary action is determined by numerous factors including the pore size and structure of the paper. The thicker, more open network allows water to wick into the paper more easily. However, at the same time, it was a challenge to observe the actual capillary flow of liquids within these papers due to the thicker and thus more opaque structure of the modified Gr17 papers compared to the Gr1 paper. In order to obtain comparable properties with Gr17 paper as with Gr1 paper, modification at RT with 2 equiv of reagent had to be applied (Table S5).

In summary, the surface properties and capillary properties of paper can be manipulated in a well-controlled manner by a range of covalent modifications via the selection of appropriate reagents with different hydrophobic tails under various reaction conditions. While previous research has focused on the wicking/nonwicking of solvents into paper in terms of simple on/off valving or extreme wettabilities,^31−33^ in contrast, our approach provides precise control over surface properties, allowing careful tuning of the paper surface properties, which can accordingly be used to broaden the applicability for on-site analytical chemistry.

#### 3.3.2 Distance-Based Analysis

In addition to surface wettability and wicking properties, paper strips were also analyzed in terms of their maximum flow distances for solutions of EtOH and water. When the modified papers were used to draw up mixtures of EtOH and water, it was observed that the resulting flow would stop after a fixed traveling distance; this occurred both when the paper was positioned horizontally or vertically, excluding gravitational pressure as the main cause. Moreover, the distance traveled by the solution after which this occurred was found to be reproducible and dependent on the % EtOH in the solution. The occurrence of a maximum flow distance has been reported by Li et al.^33^ for the hydrophobic/oleophilic paper that they obtained by fluoro-silanization, followed by oxygen plasma etching. The hypothesis to explain such a maximum flow distance is based on the evaporation of EtOH from the paper surface during the wicking process. Given that EtOH (boiling point: 78 °C) is more volatile than water, flow in an open porous system will lead to evaporation at a higher rate than the evaporation of water from the binary solutions used, effectively diluting the EtOH solution. When the % EtOH is diluted to a value at or below the CWC of the modified paper, further wicking is prevented, as previously proposed by Li et al. (Figure S14).^33^ To confirm the hypothesis, experiments were performed with the same solution-paper combinations on an open bench, in a closed chamber filled with ambient air, and in a chamber saturated with EtOH vapor. Maximum flow distances were obtained in a closed chamber filled with air with a good reproducibility (RSD = 0.8–3.5), while on an open bench the reproducibility was worse (RSD = 1.0–9.3) for C8(1)-80T6h Gr 1 paper, since the evaporation of EtOH was affected by ambient air flow (Figure S15). Moreover, the maximum distance was lower on the open bench, indicating enhanced evaporation compared to the closed system. In an EtOH-saturated chamber, on the other hand, the solutions wicked along the papers for all concentrations above the CWC until the upper end of the strips as a result of the reduced evaporation rate or the absence of net evaporation. All further analyses were therefore carried out by placing the papers vertically in EtOH solutions in the 3D-printed holder inside a closed chamber filled with ambient air (Figure S3).

In the work by Li et al., fluorinated paper was used to differentiate EtOH content of solutions with 20% concentration increments,^33^ which has limited practical value. On the other hand, in our work, the surface properties of paper were diversified over a broad range by a simple, single-step covalent modification with fatty acyl chlorides, achieving a higher sensitivity toward small increments, below 1%, of EtOH concentrations.

**Figure 3:**
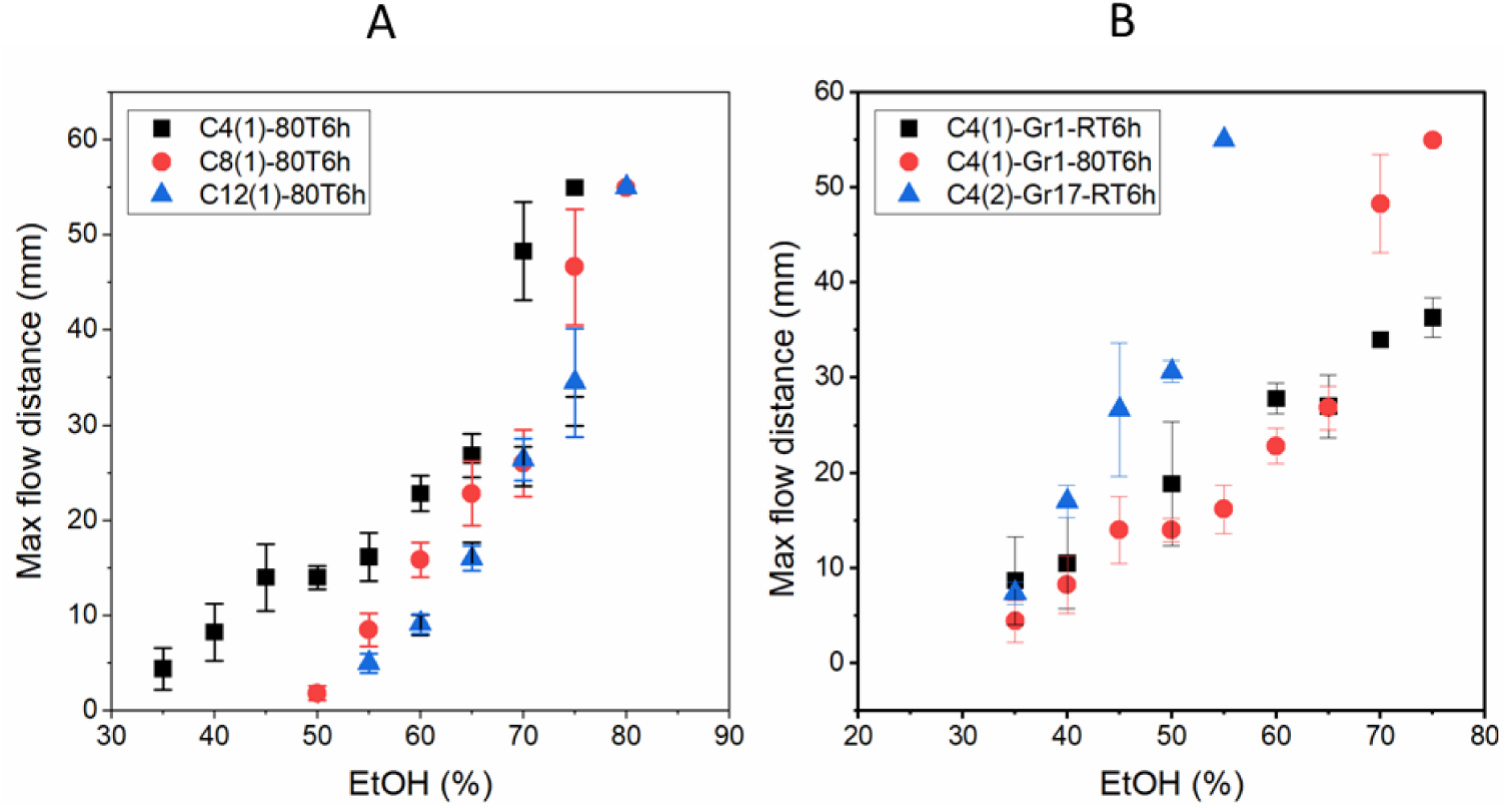
(A) Maximum flow distances measured with different water/EtOH mixtures on papers modified with different reagents (C4, C8, and C12). (B) Enhancing the sensitivity of maximum flow distance based on % EtOH with Gr1 papers modified with C4(1) at RT and 80 °C, and Gr17 paper modified with C4(2) at RT for 6 h, in which the starting CWC values of the C4(1)-Gr1–80T6h and C4(2)-Gr17-RT6h papers were the same (27% EtOH).

The maximum distance traveled by the liquid samples on modified paper was measured and correlated to the % EtOH in solution (Figure 3A). For all modified papers, the measured distance increased with the increasing % EtOH. This result was in line with expectations, because (i) the higher % EtOH in water means that the surface tension is lower, resulting in improved wettability of the hydrophobic surface, and (ii) the amount of EtOH needed to be evaporated until the solution was diluted to the CWC to stop wicking was greater, which simply takes more time.

For the % EtOH, on the other hand, the maximum distance was different for the differently modified papers. They were characterized by differences in the minimum, maximum, and total range of % EtOH that could be measured. In addition, the change in flow distance for a defined increment in EtOH concentration (Δ*d*/ΔEtOH%), which is the sensitivity of each specific paper, was evaluated as well. In Figure 3B, it was observed that Gr1 paper, both modified at RT and 80 °C using the C4 reagent, resulted in limited sensitivity. The traveling distance varied from 8 to 36 mm and 5 to 55 mm, for 35–75% EtOH, averaging 7 and 12.5 mm for every 10% change in EtOH concentration for papers modified at RT and 80 °C, respectively. However, the RT modification of Gr17 paper with the C4 reagent already resulted in a strong increase in the slope of the distance-based measurement, with the variation of traveling distance from 7 to 55 mm, for 35–55% EtOH, which results in 24 mm for every 10% change in EtOH content. It can be concluded that the sensitivity of maximum distance measurements was predominantly influenced by the specifications of the paper used rather than the reaction temperature. Nevertheless, control over both factors allowed an enhancement in sensitivity, thus demonstrating how fine-tuning of the modification can improve device performance.

As expected from WCA and CWC measurements, it was observed that with longer alkyl chain lengths of the acyl chlorides, i.e., with more nonpolar modifications, the measurement range shifted to higher concentrations (Figure 3A). This makes perfect sense, as paper needs to first wick a solution before a maximum flow distance can be observed. Furthermore, the use of more nonpolar acyl chlorides resulted in a narrower measurement range for the same observed maximum flow distance; in other words, in that range, these papers exhibit a higher sensitivity to a small change in % EtOH.

Next, the influence of the reaction temperature on the maximum flow distance was investigated. For the papers modified with C12(1), the higher reaction temperature did not affect the EtOH concentration range over which they were sensitive to changes in EtOH composition. However, the maximum distances were consistently higher for the paper modified at more elevated temperatures, thereby increasing the sensitivity to changing composition (Figure S16A). Given the fact that the hydrophobicity of the paper was not influenced for this paper, another change in the paper must have occurred. It was hypothesized that the increase in reaction temperature led to increased accessibility for the binding of the reagents due to increased swelling of the paper at higher temperatures during the reaction, and as a consequence, better coverage inside the cellulose fibers.^41^ This in turn would result in weaker interactions between the fibers, yielding a looser paper structure, which thus provides a larger surface area and thereby increases the sensitivity to changes in the solvent composition.^42^ This hypothesis was strengthened by the experiments with a particularly thick paper type, Gr17, for which a macroscopically appreciable change in structure was observed by comparing different reaction temperatures (Figure S4). In addition, it was observed that heating of the papers in solvent after modification did not have an effect on the maximum flow distance (Figure S16B); in other words, the heating during the reaction is what ultimately leads to the changes in the paper responsible for this altered wicking behavior and not the heating in the solvent by itself.

It must be appreciated that the larger the slope of the curves in Figure 3B, i.e., the higher the sensitivity to small changes in % EtOH, the more precisely and accurately such papers would be in potential distance-based sensing applications. However, especially for the somewhat more polar modifications, it was found that this slope could be very shallow (Figure 3A). Therefore, it was investigated if changing the starting paper (Gr1, Gr3MM, and Gr17), which would effectively lead to changes in the pore size, thickness, and capillary flow rates, could be leveraged to increase the sensitivity in those modified papers with limited sensitivity. According to the understanding that the maximum flow distance is determined by the evaporation of EtOH, it is clear that a higher initial flow rate would lead to an increase in the distance traveled before the concentration is diluted down to the CWC and the wicking stops. Thus, Gr1 and Gr3MM with a C8(1)-80T6h modification were tested. While no clear differences were observed in terms of maximum flow distance and sensitivity in distance-based measurements (Figure S17), clear differences were observed when Gr17 was used as the starting material and the reaction was conducted at room temperature for 6 h (Figure 3B). No distance-based data was obtained for Gr17 paper with the modifications at 80 °C for all reagents, as it led to thick paper in which flow could not be visualized (Figure S4). However, when the reaction was carried out at room temperature, the travel distance could be measured and compared with the other starting papers for the same modification (no. equiv, reaction time, and reaction temperature). In addition to that, Gr17 paper modified with 1 equiv of C4, C8, and C12 reagents did not show a CWC, i.e., they wicked water. For these modifications, the density of the hydrophobically modified groups on the paper surface could not overcome the other parameters like the porosity and larger pore size to repel water. The reagents were required to be used in higher amounts like 2 equiv for C4 and even 3 equiv for ESC to obtain a CWC value for Gr17 paper.

#### 3.3.3 Time-Based Analysis

Given the fact that it was established in previous experiments that different temperatures of the reaction affect the flow behavior, it was first assessed whether different reaction conditions, in the absence of reagents, would lead to alterations in the paper and thus result in altered flow rates. Therefore, Gr1 papers were placed in DMF, the reaction solvent, for 6 h at RT, 40 °C, and 80 °C, and then vertically run with water. The resulting flow distance was plotted against the square root of time (see eq 1, the Lucas-Washburn equation). The underlying model could be used in vertical settings, since the effect of gravity on the flow in paper was observed to be limited with these papers. It was observed that the solvent treatment had a clear impact on increasing the water flow rate in paper, while the treatment temperature did not noticeably influence the flow rate (Figure S18). Here, the Lucas-Washburn equation can be used to explain the varying parameters. First, the contact angle between the liquid and the paper is directly influenced by changes in the surface properties after modification. As introducing hydrophobic reagents to the paper surface led to a decrease in the capillary flow of water, or even rendered the paper completely impermeable to water, the surface chemistry (and thus, the resulting WCA) is the dominant factor influencing wicking and flow behavior, more so than, for example, geometrical factors (pore size and the travel distance). On the other hand, solvent treatment experiments (Figure S18) showed that geometrical changes also play a role in altering or even enhancing liquid flow. Notably, one hydrophobically modified paper, ESC(0.8)-80T6h, actually exhibited a higher water flow rate than that of bare paper. This implies that in this specific example, morphological changes had a more significant impact on the liquid flow than alterations in the surface chemistry. Ultimately, the contact angle plays a more significant role in most cases studied in this work, but the final outcome results from the combined effect of all these factors, and studying these effects in an isolated manner remains challenging.

**Figure 4:**
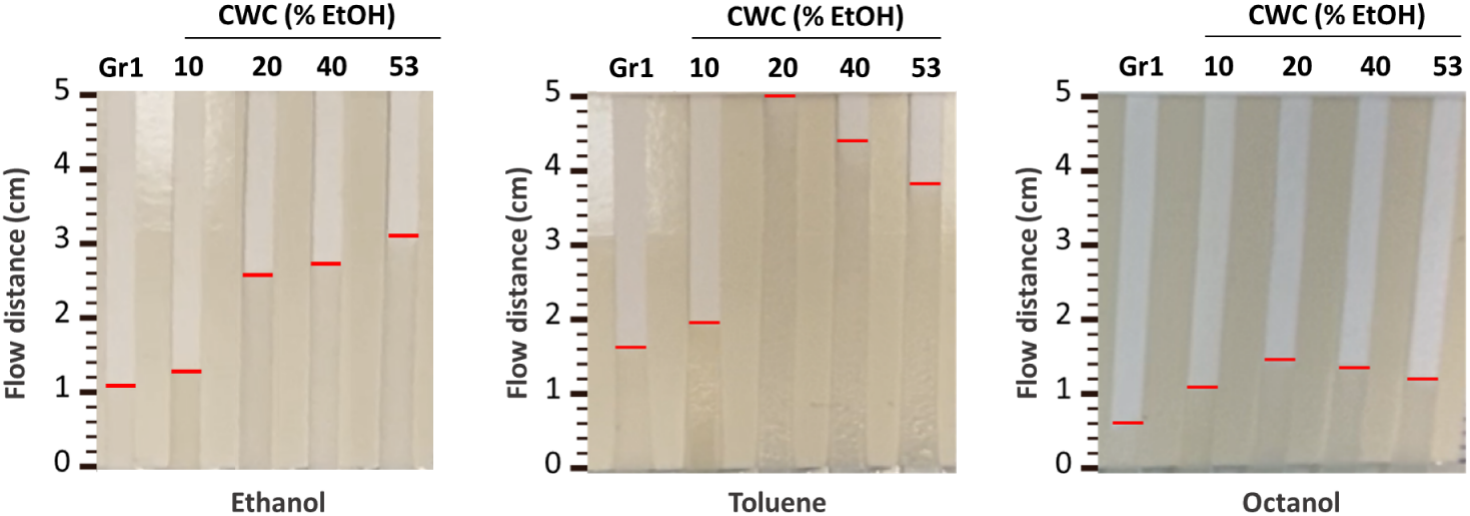
Varying flow rates of organic solvents in bare cellulose paper (positioned most left) and in the modified papers, with CWC values (in % EtOH) shown above each paper. The flow distances represent how far the liquids traveled after 40 s. Note: papers used from left to right are Gr1, ESC(1.2)-80T6h, C4(0.8)-80T6h, C8(0.8)-80T6h, and C12(1)-80T6h.

In addition, while most paper microfluidic devices are operated with aqueous solutions, it is often beneficial to be able to work with less polar organic solvents as well, for example, to improve selectivity by liquid–liquid extraction or to work with less-polar analytes. However, regular cellulose paper might not be the most suitable material to allow the capillary flow for organic solvents. To gain a better insight into that, the flow of ethanol, toluene, and octanol within five different papers was monitored, namely, bare cellulose and modified papers with varying CWCs of 10%, 20%, 40%, and 53% EtOH. The flow rate of all solvents was slowest on bare cellulose paper due to the weak interaction between the paper surface and the solvent. In contrast, the highest flow rate for octanol and toluene solvents was achieved on C4(0.8)-80T6h paper with a CWC of 20% EtOH, and the flow rate kept decreasing on papers with a higher CWC. On the other hand, ethanol flowed faster in more hydrophobic papers, and it reached the highest flow rate on C12(1)-80T6h paper. The overall lower flow rates of octanol compared to ethanol and toluene are due to its higher viscosity (dynamic viscosities of 0.59, 1.2, and 9.27 mPa·s at 20 °C for toluene, ethanol, and octanol, respectively; Figure 4).^43−45^ Critically, this result shows that hydrophobic modifications for paper-based systems working with organic solvents are important to optimize flow and that tuning the surface modification allows for fine control also of nonaqueous solutions.

Finally, water-permeable modified papers were used to compare their water and EtOH flow rates with those of bare grade 1 paper (Figure S19). Water flowed faster in ESC(0.8)-80T6h and slower in C16(0.4)-80T6h papers compared with unmodified hydrophilic paper. While it is true that, compared to unmodified paper, ESC(0.8)-80T6h paper was more hydrophobic, it was also subjected to the modification process in DMF at 80 °C, which resulted in changes to the structure of the paper, thereby influencing the flow rate. On the other hand, the flow of EtOH was the slowest on unmodified paper and the fastest with ESC(0.8)-80T6h paper. This result implies that the combination of polar and nonpolar interactions between the surface and the solvent, and the physical changes that occurred in the paper structure due to the modification resulted in the fastest flow.

### 3.4 Applications

The aim of developing well-controlled wettability and capillary flow in a wide range of hydrophobically modified papers has been to enable their implementation in paper microfluidic devices with improved functionality. Here, we demonstrate the applicability of these modified papers by employing their controllable permeability, maximum flow distance, and flow rates for various solvents. These proof-of-concept demonstrations include: (i) permeability-based surface tension measurement, (ii) permeability-based valving in multistep colorimetric nitrite detection, (iii) maximum flow distance-based alcohol sensing, and (iv) optimization of capillary flow rates for paper-based liquid–liquid extraction (LLE).

#### 3.4.1 Permeability-Based Sensing

**Figure 5:**
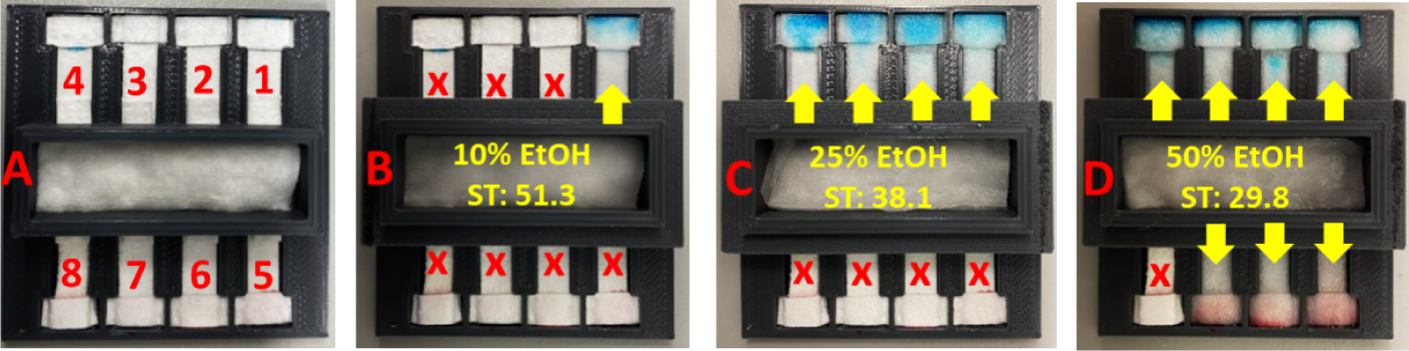
(A) Permeability-based surface tension measurement device. Modified papers in the channels have CWC of 6%, 12%, 17%, 22%, 36%, 42%, 49%, and 52% EtOH corresponding to positions 1 through 8, respectively. Applied aqueous EtOH solution concentrations: (B) 10%, (C) 25%, and (D) 50%, where ST stands for surface tension at 20 °C (mN m^–1^). Note: the papers used from positions 1 to 8 PAC(1), PAC(1.2), C4(0.8), POAC(1.2), C6(0.6), C6(1), C16(0.7), and C12(1) were modified at 80 °C for 6 h.

Eight modified papers with CWCs that varied between 6 and 52% were selected to conduct permeability-based surface tension measurements using EtOH solutions of varying surface tensions. A 3D-printed holder was designed with a sample reservoir in the middle and separate channels to place papers of varying CWC (Figures 5A; see S20 and S21 for details of the 3D design and device assembly). A drop of blue (top part of the device, EtOH concentrations of 10–25%) or red (bottom part of the device, EtOH concentrations of 40–55%) food dye solution was preloaded to the outer edge of the modified paper strips to create colorimetric detection points. Those colored spots were then covered with a piece of unmodified paper; only when the modified paper with dye would be wetted—if the sample EtOH content exceeded the threshold (CWC)—would the dye be transported onto the top paper, making the color visible. This experiment was performed with 5% increments in EtOH concentration, and these were successfully differentiated from each other due to their differences in surface tensions. For example, the color only appeared at the detection point of the first channel as a result of using a 10% EtOH solution (Figure 5B), which had higher alcohol content than the CWC of PAC(1) paper but less than the other papers, while 25% EtOH was required to produce a color change in the first four detection points (Figure 5C) and 50% for all but the last pad (Figure 5D). Figures for all eight reading spots for eight different EtOH solutions can be found in Figure S22.

#### 3.4.2 Permeability-Based Multistep Valving

The selective flow properties of hydrophobic papers were further leveraged in a multistep on/off valving system for colorimetric nitrite detection. Nitrite is a toxic compound that is harmful to human health and the environment. The European Commission’s former Scientific Committee for Food (SCF) and the Joint FAO/WHO Expert Committee on Food Additives (JECFA) established the current acceptable daily intakes (ADIs) for nitrite in 1997 and 2002. These ADIs, which are measured in milligrams per kilogram of body weight per day (mg/kg bw/day), were 0.06 and 0.07, respectively. It is therefore allowed below certain limits, for example, in drinking water below 1 ppm,^46^ and as a preservative and color fixative in finished meat products below 200 ppm.^47^ Colorimetric detection can be achieved via the multistep Griess reaction (Figure S23), which should be performed sequentially to yield the azo-compound that gives a strong purple color response in the presence of nitrite. In the first step, nitrite reacts with sulfanilamide (SA) to produce diazonium salt (Figure S23A), and then, this intermediate product reacts with naphthylethylenediamine (NED) to produce the azo-compound that results in a color change from colorless to purple (Figure S23B).^48^ The compounds SA and NED are, in the device below, deposited on different paper pads bearing their abbreviated name.

**Figure 6:**
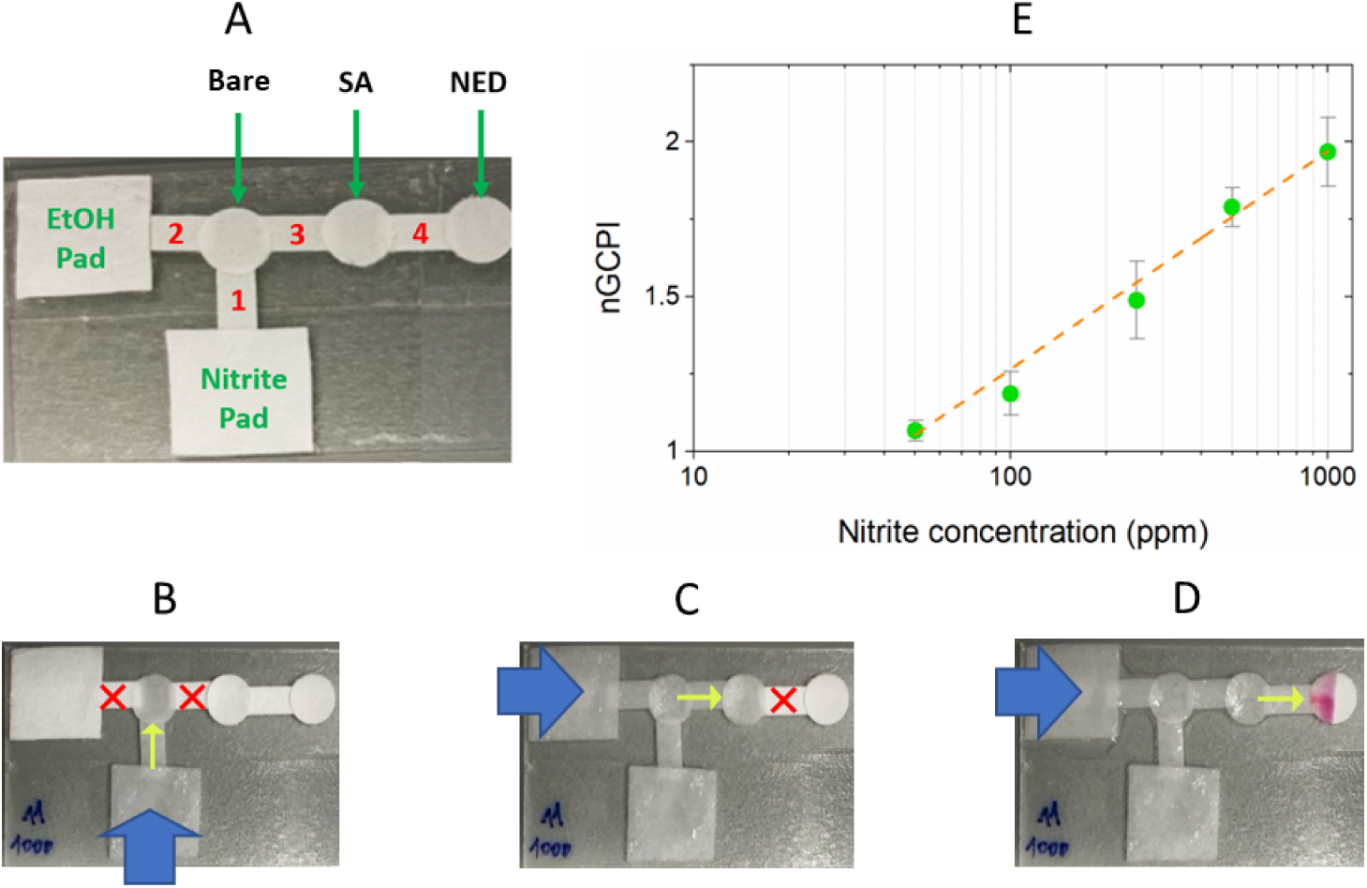
(A) Permeability-based multistep valving device for nitrite detection. Circular papers are bare Gr1 papers or preloaded with 2 μL of 2% SA or 2 μL of 3% NED. Rectangular papers are (1) bare Gr1 for nitrite collection, (2) and; (3) C4(0.8)-80T6h papers with the CWC of 20% EtOH to prevent the flow of aqueous sample solution, and (4) C12(1)-80T6h with the CWC of 52% EtOH to stop flow after the first reaction step. (B) When aqueous nitrite solution is applied to the pad, it travels through **1**, but is stopped at **2** and **3** (see the red cross, **×**, signs) to entrap and enrich the nitrite in the bare circular paper. (C) When a first EtOH aliquot is added on the EtOH pad, **2** and **3** can be wetted and allow controlled transfer of nitrite from the bare paper pad to the SA paper pad for the first step of the Griess reaction. (D) When subsequently a second EtOH aliquot is added, **4** will also be wetted and the reaction product is transported from the SA paper to the NED paper for the second reaction step of the Griess reaction to obtain a color change. (E) After the color appeared, the normalized green color pixel intensity (nGCPI) was plotted against the nitrite concentration (*n* = 3, error bars represent standard deviation).

A paper-based multistep valving device, shown in Figure 6A, was thus designed and assembled for this application (Figures S24; see S25 for the schematic flow of multistep valving). First, the color responses of different reaction sequences were investigated to confirm that the correct sequence of reactions gives the strongest color response on paper; the device was then tested ( Figure S26A–C). After applying 50 μL of aqueous nitrite solution to the nitrite sample pad, the aqueous sample would reach the first circular bare paper through paper 1. Here, the flow stopped, as papers 2 and 3 (C4 (0.8)-80T6h) would not wick water; these valves were thus “off” (Figure 6B). When 50 μL of EtOH was then loaded onto the EtOH pad, the surface tension of the aqueous solution would decrease to allow the transfer of nitrite to the SA paper pad; the valves “2” and “3” would be “on” (Figure 6C). Still, the last valve, paper 4 (C12 (1)-80T6h), remained “off” as it had a higher CWC than papers 2 and 3. Thus, the nitrite reacted at the SA location to produce the diazonium salt product. After 1 min, another 50 μL of EtOH was applied onto the EtOH pad to further reduce the surface tension of the wicking solution and open the last valve, thus allowing the diazonium salt to move to the NED paper for the second step of the Griess reaction and produce the azo-compound that gave the purple/pink color response (Figure 6D). After obtaining a color change in the detection zone for varying concentrations of nitrite (Figure S26D), the green color pixel intensity (GCPI) was measured by using the software ImageJ 1.52.^49^ Due to the inverse relationship between pixel intensity and actual color intensity (the pixel intensity decreases as the color intensity increases), normalization of pixel intensities was carried out by dividing the blank solution’s value by the GCPI values of the nitrite solutions. These values were then plotted against the nitrite concentration (Figure 6E). As can be seen, a linear correlation was obtained, showing that the use of appropriately modified paper pads allows the determination of a relevant analyte simply by the two-time addition of an EtOH solution.

#### 3.4.3 Distance-Based Sensing

**Figure 7:**
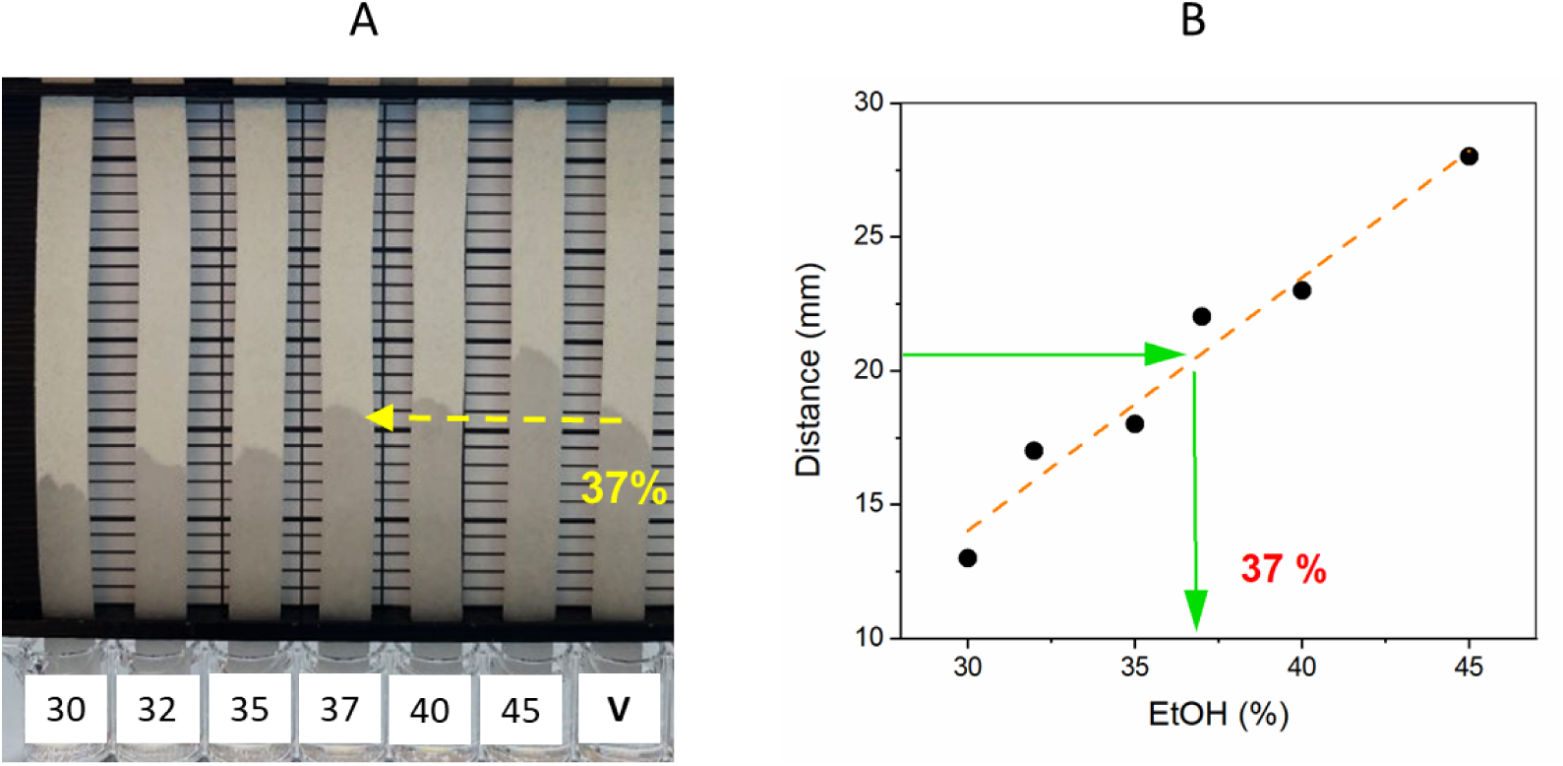
Distance-based alcohol sensing using a series of aqueous EtOH solutions (with % EtOH ranging from 30% to 45%) to determine the % EtOH in vodka (data point V), claimed to be 37.5% using the maximum flow distance, measured (A) visually and (B) after image analysis. Note: C4(1)-80T6h paper with CWC of 27% EtOH was used throughout.

The maximum flow distance of modified papers was then applied for the alcohol determination in a commercial alcoholic beverage, namely, vodka with 37.5% v/v alcohol content, as reported by the producer. C4(1) paper was used, as well as reference solutions in the concentration range of the sample’s alcohol content (30%, 32%, 35%, 37%, 40%, and 45% EtOH solutions; Figure 7A). The maximum distance traveled by the sample and the references was then compared to determine the alcohol content of the beverage. The concentration was determined through two methods: visual inspection using the naked eye and analysis of a graph plotted from the data points obtained. The gridlines in the 3D-printed device indicated a concentration of 37% when observed visually with the naked eye (Figure 7A), which was confirmed with the graph generated from the sample and reference solutions’ data points (Figure 7B).

#### 3.4.4 Flow/Timing Control

**Figure 8:**
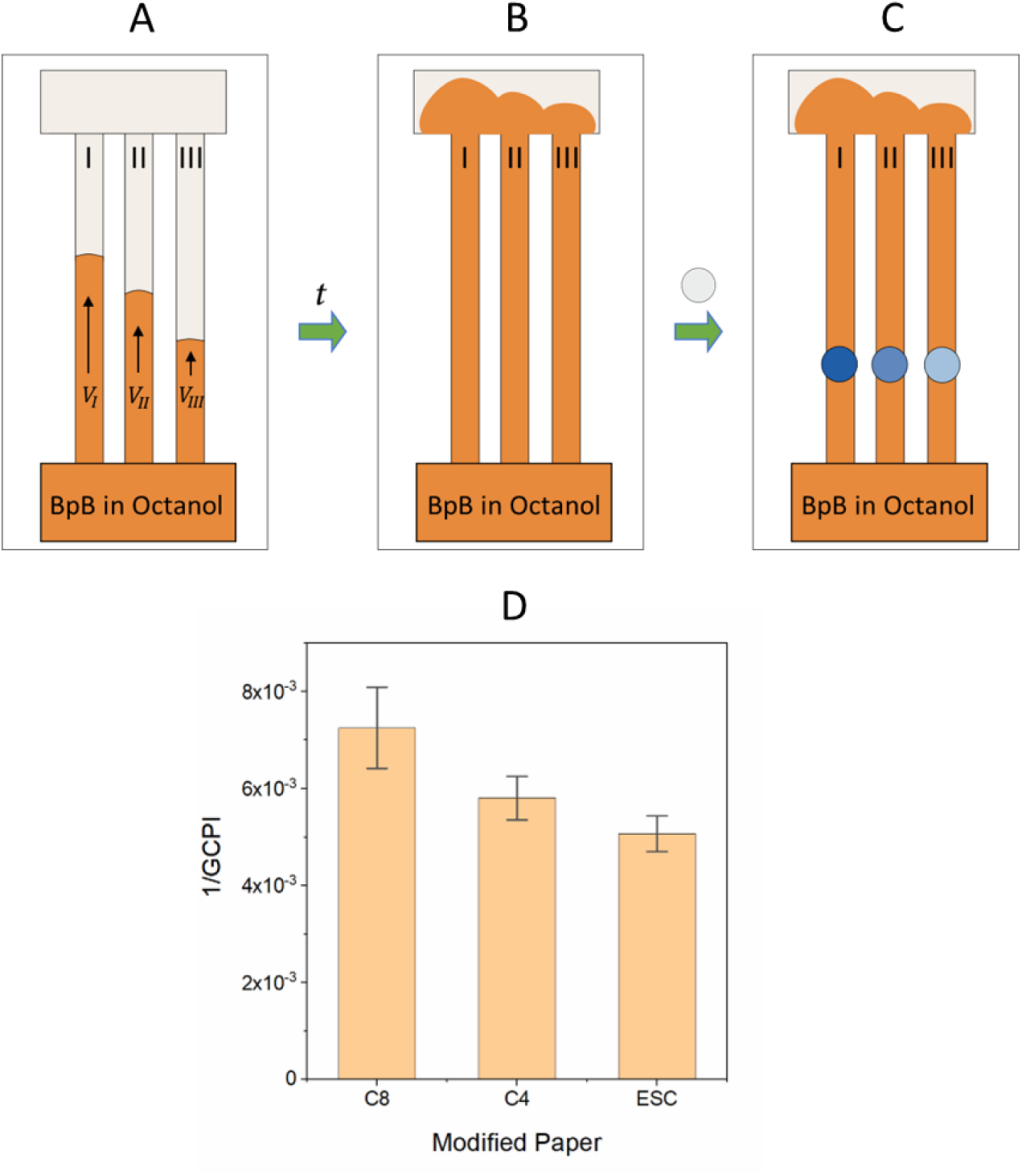
Paper-based LLE efficiency optimization scheme: (A) 0.2% w/v BpB in octanol (orange color) flows through modified papers with divergent CWC of (I) 10%, (II) 27%, and (III) 47% EtOH, having velocities in the following order *V*_I_ > *V*_II_ > *V*_III_; (B) when they reach the absorption pad (C) circular Gr1 papers prewetted by water were placed on the paper strips for 30 s to allow the transfer of BpB from octanol (nonpolar) phase to the water (polar) phase, which then turns blue, after which they were removed and dried under ambient conditions. (D) The inverse GCPI results for each modified paper are in decreasing order with decreasing flow rate. Note: papers used were (I) C8(1)-80T6h, (II) C4(1)-80T6h, and (III) ESC(1.2)-80T6h.

The control over flow rates through hydrophobic papers allows for optimizing the efficiency of, e.g., paper-based liquid–liquid extraction (LLE).^50^ Solvents flow at varying speeds on differently modified papers, as clearly shown in a time-based analysis (Figure 4). This property was studied to observe the variation in the mass transfer rate of bromophenol blue (BpB) from octanol to water via paper-based LLE by using different hydrophobically modified papers. Experiments were performed by placing circular bare papers, prior to being soaked in water, on paper strips taped on a backing card, on which octanol with BpB flows (Figure 8A–C). Following their removal after 30 s, the papers were dried, and the GCPI was determined using ImageJ. To demonstrate the direct relationship between the color intensity and concentration (darker color indicates more BpB passed to the water phase), the inverse values of the GCPI were used to establish a correlation. This serves as an indication of the amount of BpB on the circular papers after the LLE. Since the exact amounts of transferred BpB were not known, inverse values of GCPI were taken as an indication without further normalization (Figure 8D).

The experimental findings indicate that an increase in the flow rate leads to a corresponding increase in extraction efficiency; higher flow rates facilitate a greater convective mass transfer of the analyte from one phase to another. Consequently, these modifications demonstrate the potential for optimizing such systems by effectively pairing the (modified) paper with the appropriate solvent in paper microfluidic devices, thus achieving enhanced performance. Conversely, since the opposite is also valid as the increasing mass transfer is caused by increasing flow rate, this feature can be simply used to obtain insight into quasi-steady flow through paper-based systems, which applies in the case of using absorbent pads.^51^

## 4 Conclusions

In this research, a family of simple covalent modifications has been studied and applied to manipulate the surface properties of paper toward several μPAD applications. The potential of covalent paper modifications to develop tunable on-site sensing platforms for a wide range of analytes in different matrices was investigated by systematic variation of various properties of the modified papers, namely permeability (wicking/nonwicking), maximum flow distance, and flow rate. It was demonstrated that the typical (and often critical) lack of control over the flow in μPAD applications can be overcome by such easy and highly tunable chemical modifications. Various proof-of-concept devices that hinge on the resulting fine-tuned flow properties were demonstrated, enabling easy sensing and actuating for improved on-site analysis. The wide range of examples that were included (measurement of the surface tension of a solution based on maximum flow distance, determination of the EtOH content of alcoholic beverages, solvent-dependent valving, permeability-based surface tension sensing, and optimization of paper-based liquid–liquid extraction using timing control) show the significant potential of these simple covalent modifications of cellulose paper. Ongoing work in our laboratories focuses on further fine-tuning of the attached layers and an extension to more advanced devices for PoC and PoN analysis.

## Supporting Information Available

Additional details on the results and analyses, including acylation reaction mechanism, CWC and WCA values of modified papers, ATR-FTIR spectra for bare and modified papers, additional results on distance- and time-based experiments to assess the effect of different reaction parameters, design details of 3D-printed holders, their assembly processes, schematic details of application devices (PDF)
