# Supplementary material for: Broad Tuning of Paper Microfluidic Properties by Covalent Surface Modification for Precise Flow Control and Sensing
Source: ACS Appl Bio Mater. 2025 Apr 17;8(5):3748–61. doi: 10.1021/acsabm.4c01812 (PMC12093371; doi:10.1021/acsabm.4c01812)
Supplement: Supplementary file 1 — mt4c01812_si_001.pdf [file mt4c01812_si_001.pdf]

Supporting Information

Broad Tuning of Paper Microfluidic Properties by Covalent Surface Modification for Precise Flow Control and Sensing

Canan Aksoy[1,2], Ischa van Kesteren[1], Han Zuilhof[1,3], Gert IJ Salentijn[1,2,*]

[1] Wageningen University, Laboratory of Organic Chemistry, Helix Building 124, Stippeneng 4. 6708 WE Wageningen, The Netherlands.

[2] Wageningen Food Safety Research, Wageningen University and Research, P.O Box 230, 6700 AE Wageningen, The Netherlands.

[3] College of Biological and Chemical Engineering, Jiaxing University, Jiaxing 314001, China.

* Corresponding author: gert.salentijn@wur.nl

Table of Contents

Table S1	Specifications of the chromatography papers used	Page S4
Figure S1	Acylation reaction mechanism for the covalent hydrophobic modification of cellulose paper	Page S4
Table S2	N-octanol-water partitioning coefficients of reagents	Page S4
Table S3	Acylation reaction equivalence constants of reagents calculated with equation S1	Page S5
Equation S1	Equation for the reagent amount based on the desired equiv.	Page S5
Table S4	Overview of the modifications for various reaction conditions, variable papers, different reagent types and amounts	Page S6
Figure S2	Determining the changes in wettability and critical wicking concentration (CWC)	Page S7
Figure S3	3D-printed vertical flow test device for distance- and time-based analysis.	Page S8
Figure S4	Bare (unmodified), C8(1)-RT6h and C8(1)-80T6h Gr 17 papers	Page S9
Figure S5	ATR-FTIR spectra of modified Gr 1 papers with different chain length of fatty acyl chlorides at 80°C for 6 h and bare paper	Page S9
Figure S6	ATR-FTIR spectra of Gr 1 paper modified with ESC(1.2), PAC(1.2) and POAC(1.2) at 80°C for 6 h and bare paper	Page S10
Figure S7	ATR-FTIR spectra of Gr 1 paper modified with C16(2) at 80°C for reaction times of 1, 2, 4 and 6 h	Page S10
Figure S8	ATR-FTIR spectra of Gr 1 paper modified with C8(2) at 80°C for reaction times of 1, 2, 4 and 6 h	Page S11
Figure S9	ATR-FTIR spectra of Gr 1 paper modified as C4(1)-80T6h, C6(1)-80T6h, C8(1)-80T6h and C12(1)-80T6h	Page S12
Figure S10	ATR-FTIR spectra of Gr 1 paper modified with C8(1) at RT, 40 and 80°C for 6 h	Page S13
Figure S11	ATR-FTIR spectra of Gr 1, Gr 3MM and Gr 17 papers modified with C8(1) at 80°C for 6 h	Page S13
Figure S12	ATR-FTIR spectra of modified papers treated in varying pH levels	Page S14
Table S5	Water contact angle (WCA) and CWC critical wicking concentration (CWC) values of covalently modified papers for various reagents, starting papers and different reaction conditions	Page S15
Figure S13	Scanning Electron Microscope (SEM) images of bare grade 1 paper, DMF-treated bare paper at RT, DMF-treated bare paper at 80°C, and C16(0.6)-80T6h paper	Page S16
Figure S14	Proposed explanation for the occurrence of a maximum flow distance in modified papers, and an example of increasing maximum flow distances with increasing EtOH content	Page S17

Table of Contents (continued)

Figure S15	Maximum flow distance measurement of C8(1)-80T6h on an open bench and in an air saturated closed chamber to investigate the repeatability of the measurements	Page S17
Figure S16	The effect of reaction temperature on maximum flow distance for C8(1) paper modified at different temperatures: RT, 40ºC and 80ºC; the effect of treating C8(1)-RT6h papers in DMF at 40ºC and 80ºC for 6 h after modification	Page S18
Figure S17	Maximum flow distances of C8(1)-80T6h Gr 1 and Gr 3MM papers	Page S18
Figure S18	Distance travelled as a function of square root of time of 50% EtOH solution in untreated Gr 1 paper, and the treated papers in DMF at RT, 40ºC and 80ºC for 6 h	Page S19
Figure S19	Varying flow rates of water and ethanol in modified papers	Page S19
Figure S20	SolidWorks drawings of 3D printed permeability-based sensing device with its dimensions: isometric and dimetric views of the additional part; isometric and top views of the main device	Page S20
Figure S21	Assembly of the permeability-based sensing device	Page S21
Figure S22	Permeability based surface tension measurement device	Page S22
Figure S23	Multistep colorimetric Griess reaction	Page S23
Figure S24	Assembly of permeability-based multistep valving device on a piece of adhesive backing card for nitrite detection	Page S24
Figure S25	Schematic flow of multistep valving for nitrite determination	Page S25
Figure S26	Color response of different reaction sequences; Color response of various concentrations of NO_2^- aqueous solution from the multistep colorimetric Griess reaction	Page S26
References		Page S26

Table S1. Specifications of Whatman® chromatography papers used

Type of paper	Thickness (mm)	Flow rate (mm/30min)*
Grade 1	0.18	130
Grade 3MM	0.34	130
Grade 17	0.92	190

*reported by the manufacturer

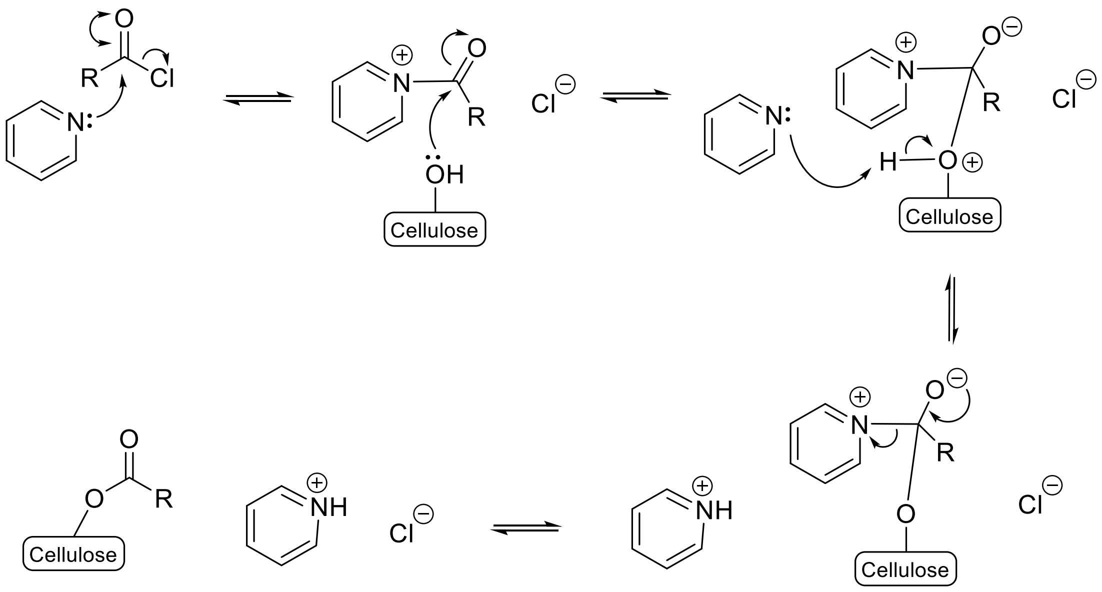

Figure S1. Acylation reaction mechanism for the covalent hydrophobic modification of cellulose paper[1]

Table S2. N-octanol-water partitioning coefficients of reagents (from top to bottom in an increasing order), which were obtained from ChemDraw 20.1.1 software

Compound	logP
Ethyl succinyl chloride, ESC	0.54
Butyryl chloride, C4	1.34
Phenoxy acyl chloride, POAC	1.75
Phenyl acyl chloride, PAC	2.11
Hexanoyl chloride, C6	2.17
Octanoyl (Caprylyl) chloride, C8	3.01
Dodecanoyl (Lauroyl) chloride, C12	4.68
Hexadecanoyl (Palmitoyl) chloride, C16	6.35

Table S3. Acylation reaction equivalence constants of reagents calculated with equation (S1).

**Compound**	**Equivalence Constants**
Pyridine	1.50
ESC	2.64
POAC	2.56
PAC	2.45
C4	1.97
C6	2.49
C8	3.16
C12	4.28
C16	5.62

Equivalent (equiv.) amounts of reagents and pyridine were calculated with eq **Error! Reference source not found.**S1, where three OH groups are available in one anhydroglucose unit (AGU). In order for easy calculation of the amount of reagents and pyridine needed for a reaction, equivalence constants were calculated for 1 equiv. amount for every reagent (SI, Table S3). An equivalence constant was defined as a multiplication factor for easy calculation of the required amount of reagents in the desired modification. The amount was simply calculated by multiplying the corresponding equivalence constant with the mass of cellulose paper used in the modification and the desired number of equivalents of the reagent.

$$V_{reagent}(ml) = \frac{m_{cellulose}(mg)\times 3}{MW_{AGU}} \times \frac{MW_{reagent}}{\rho_{reagent}\ (mg/ml)} \times equiv._{reagent} \qquad (S1)$$

Table S4. Overview of the modifications for various reaction conditions, variable papers, different reagent types and amounts

**Paper**	**Reagent**	Equiv.	**Time (h)**	**Temperature (ºC)**
Gr 1	ESC	0.8*, 1, 1.2, 1.5	6	80
	PAC	0.8*, 1, 1.2, 1.5	6	80
	POAC	0.8*, 1, 1.2, 1.5	6	80
	C4	0.7*, 0.8, 1, 1.2, 1.5	6	80
		1	16, 24	80
		1	6	RT, 40
	C6	0.5*, 0.6, 0.8, 1	6	80
	C8	0.5*, 0.6, 0.7, 0.8, 1, 2	6	80
		2	1, 2, 4	80
		1	6	RT, 40
	C12	0.4*, 0.5, 0.6, 1, 2	6	80
		1	16, 24	80
		2	1, 2, 4	80
		1	6	RT, 40
	C16	0.3*, 0.5, 0.6, 0.7, 0.8, 1, 2	6	80
		2	1, 2, 4	80
Gr 3MM	C8	1	6	80
Gr 17	C8	1*, 2**	6	80
	C4	1*, 2**	6	40, 80
		2	6	RT
	ESC	3	6	RT
	C12	2	6	RT, 40, 80

*represents the threshold for equiv. amounts of the reagents in the corresponding reaction conditions to become impermeable to water. Including and below the given equiv. values, modified papers were permeable to water.

**represents highly swollen papers after the modification, where the high opacity originated from the thickness of the modified paper, which limited our ability to observe the flow of liquid in paper by eye.

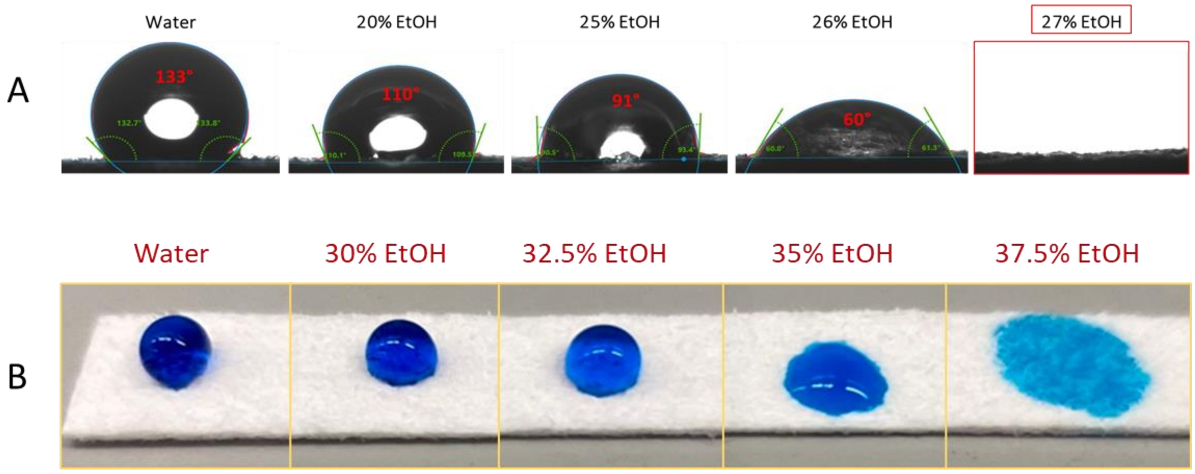

Figure S2. Determining the changes in wettability and critical wicking concentration (CWC). The experiments were conducted in two ways: (A) 3 µL droplets of solutions were placed on modified papers with the syringe in the dispensing system of contact angle measurement device, and CWC values were recorded (C4(1)-80T6h paper was used in this example) and (B) 5 µL droplets of solutions (blue food dye is used for better visualization) were dropped on modified papers (C8(0.8)-80T6h paper was used in this example); then the CWC values were recorded as the lowest wicking concentration of EtOH solutions in the same manner as in (A).

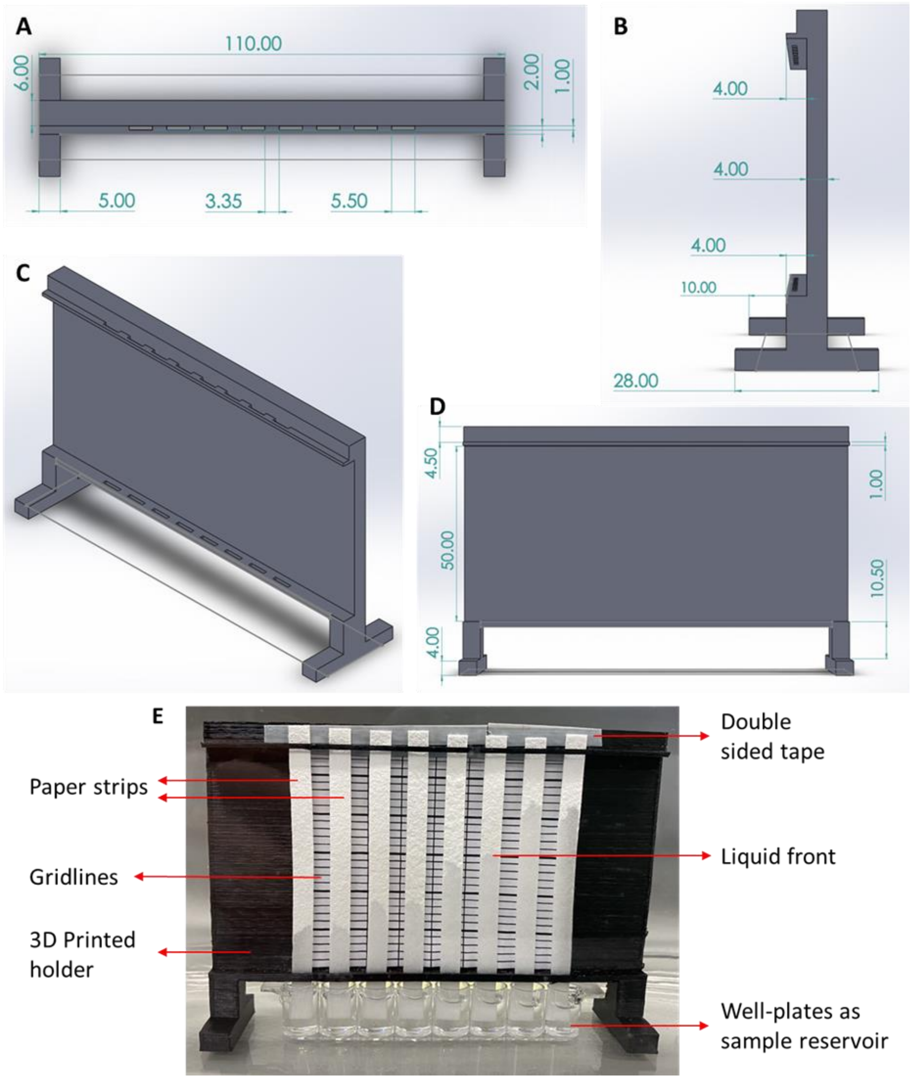

Figure S3. 3D-printed vertical flow test device for distance- and time-based analysis: SolidWorks drawings with dimensions (in mm) from (A) top, (B) side, (C) isometric and (D) front views, and (E) the holder front view in use. The 3D-printed holder was designed to meet a number criteria to develop a facile and robust method for measuring flow through paper strips. It needed to have a rigid structure, allow running multiple strips in parallel, have a good alignment of papers, avoid contact of paper with the device material not to have any interference with the flow, protect strips from the surroundings (e.g. air flow), which influences the evaporation of ethanol from the paper surface, and have gridlines to facilitate accurate data acquisition. Upper and lower holes were used for alignment of papers with each other and creating a space behind the papers; double sided tape was used to attach papers to the holder; the height of the bottom holes was designed to allow easy suspension of the attached papers into the well-plate below, filled with the solutions. Gridlines were prepared and printed in 2 mm increments to facilitate accurate data acquisition. The bottom holes were aligned with the grid and served as reference line for the start of the measurements.

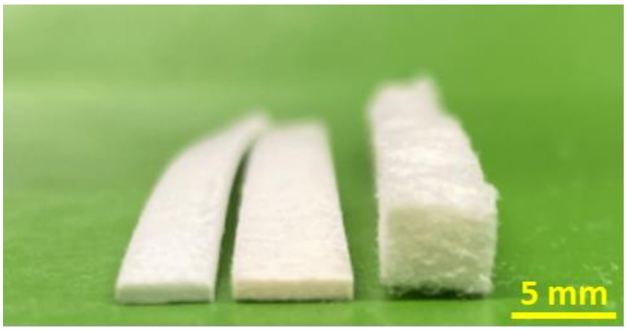

Figure S4. Bare (unmodified), C8(1)-RT6h and C8(1)-80T6h Gr 17 papers (from left to right)

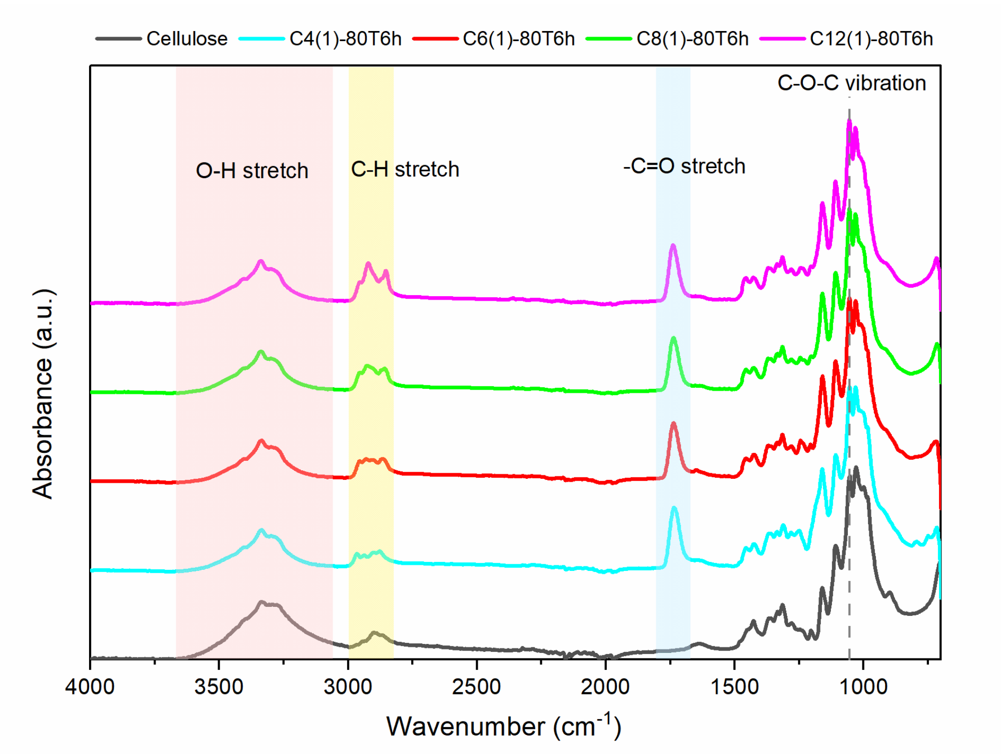

Figure S5. ATR-FTIR spectra of modified Gr 1 paper with different chain length of fatty acyl chlorides (1 equiv., C4, C6, C8, C12) at 80°C for 6 h and bare paper

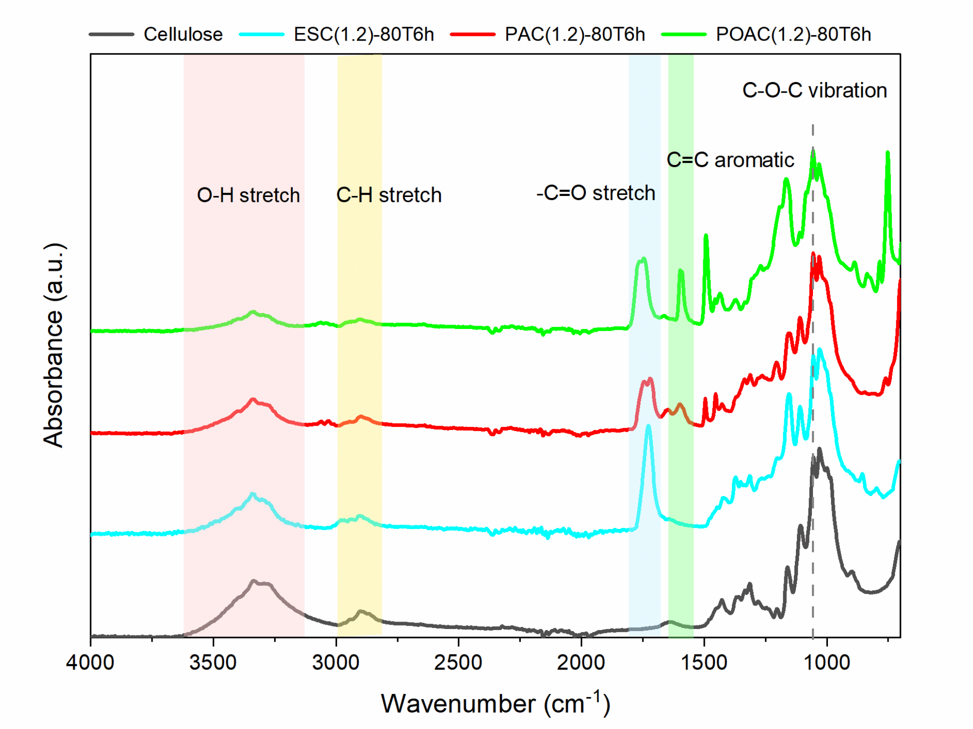

Figure S6. ATR-FTIR spectra of Gr 1 papers modified with ESC(1.2), PAC(1.2) and POAC(1.2) at 80°C for 6 h and bare paper

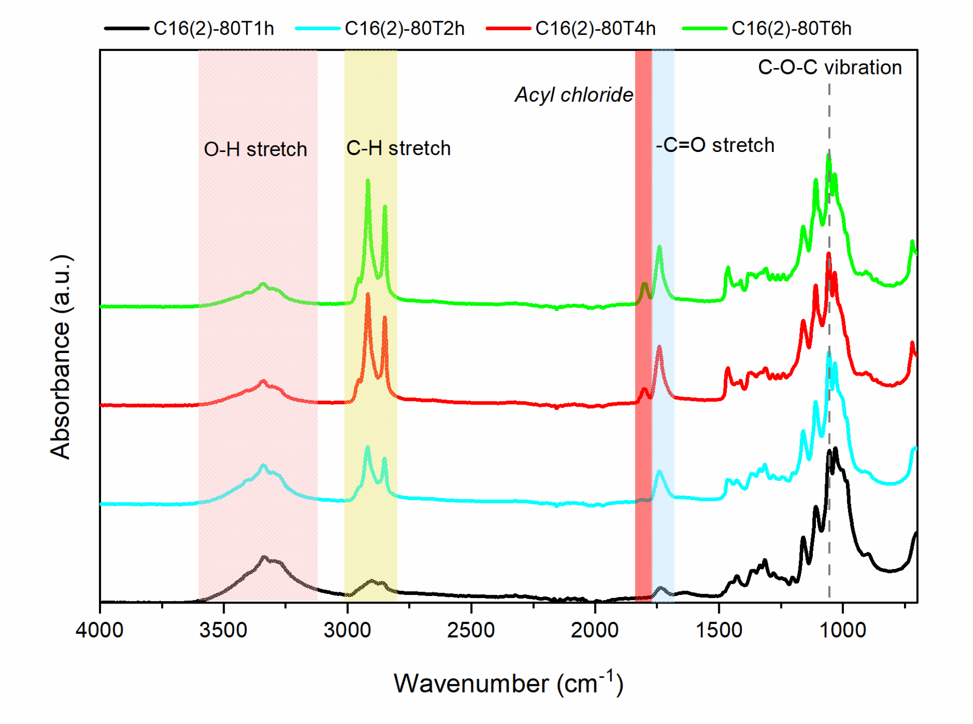

Figure S7. ATR-FTIR spectra of Gr 1 paper modified with C16(2) at 80°C for reaction times of 1, 2, 4 and 6 h

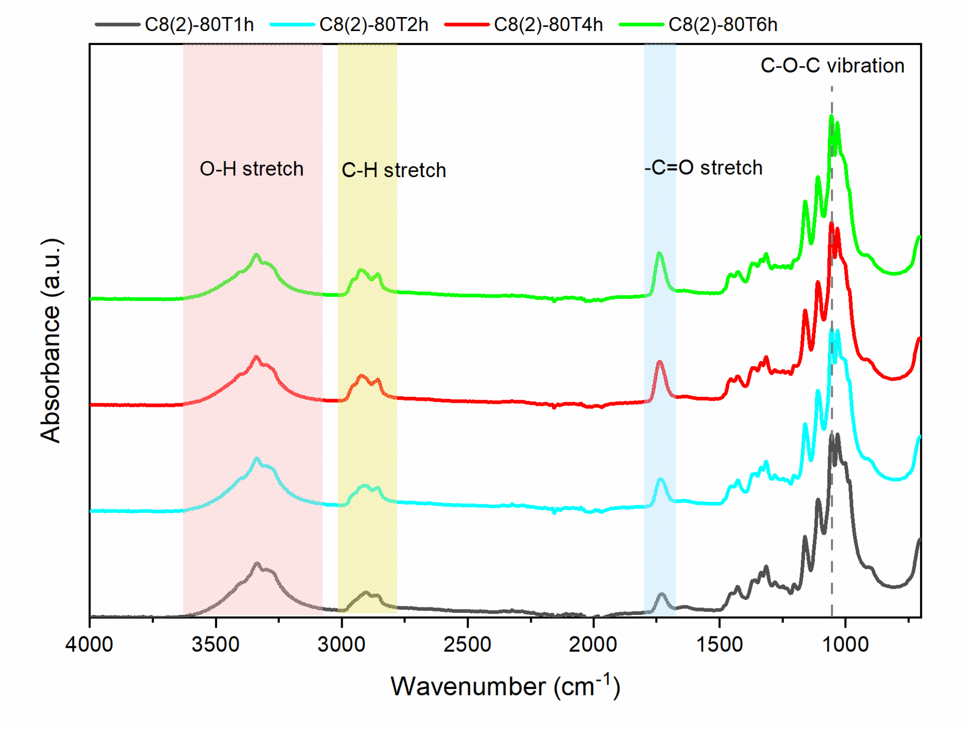

Figure S8. ATR-FTIR spectra of Gr 1 paper modified with C8(2) at 80°C for reaction times of 1, 2, 4 and 6 h

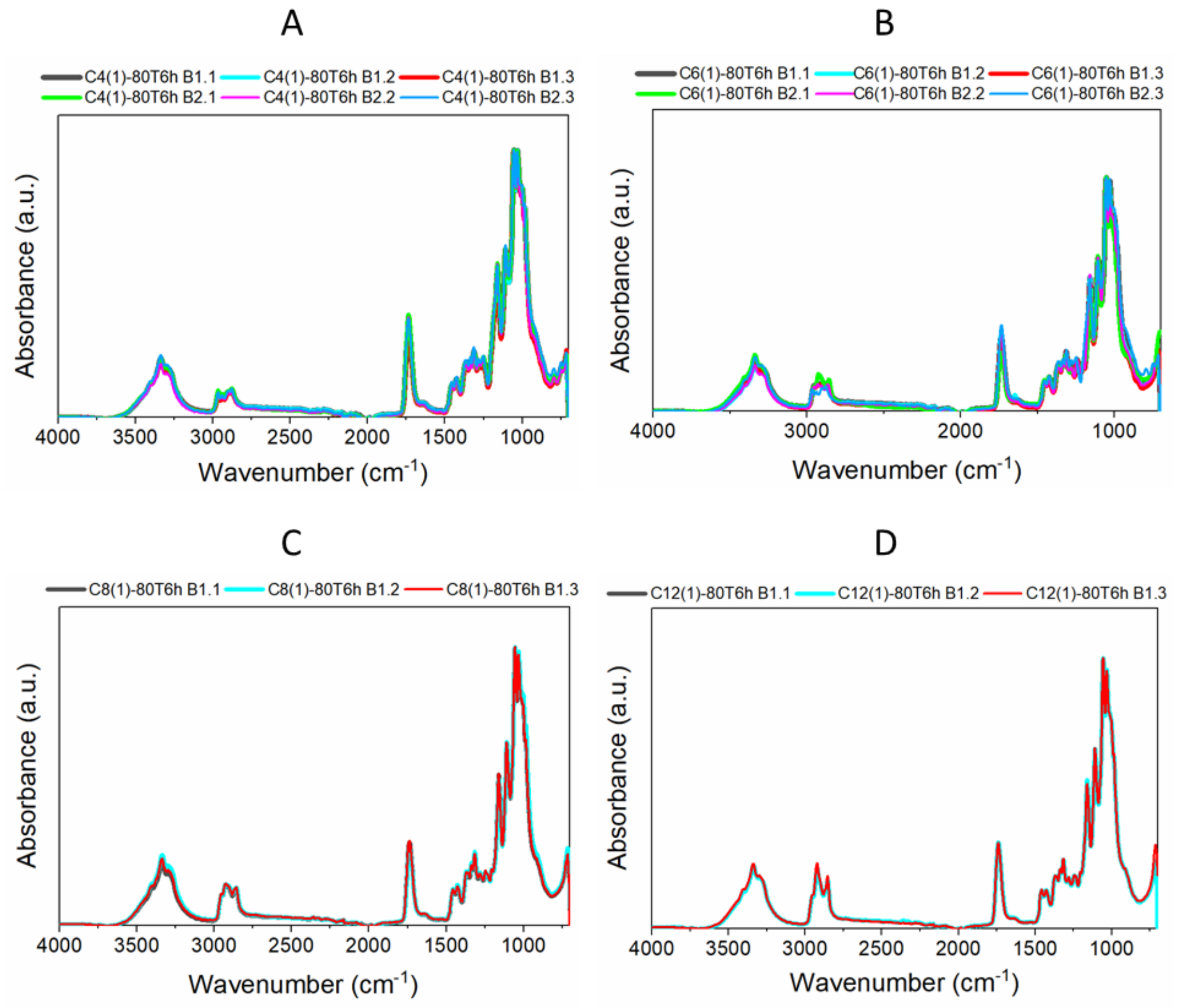

Figure S9. ATR-FTIR spectra of Gr 1 papers modified as (A) C4(1)-80T6h, (B) C6(1)-80T6h, (C) C8(1)-80T6h and (D) C12(1)-80T6h, where B1 and B2 represents two equally treated modified papers from different batches, and B1.1, B1.2, and B1.3 represents measurement from three locations on the same strip

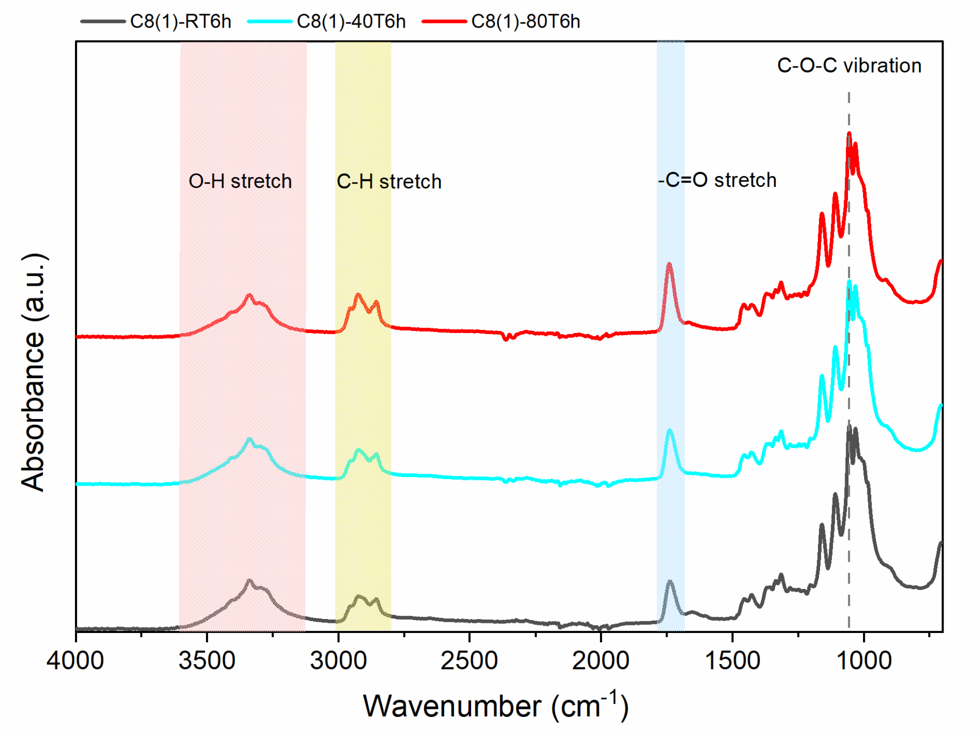

Figure S10. ATR-FTIR spectra of Gr1 papers modified with C8(1) at RT, 40 and 80°C for 6 h

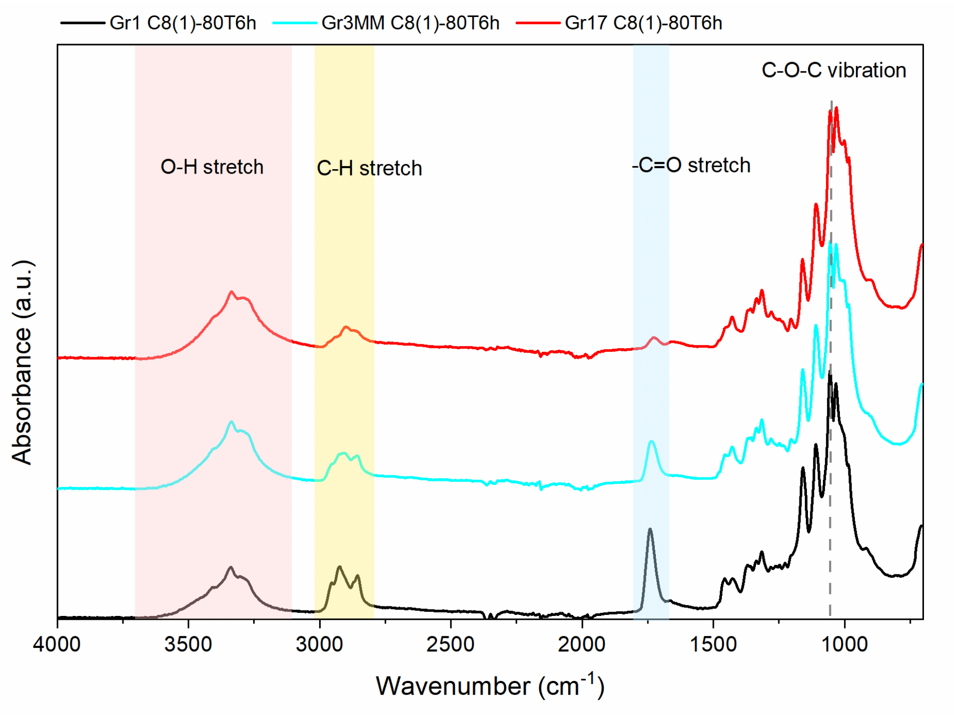

Figure S11. ATR-FTIR spectra of Gr 1, Gr 3MM and Gr 17 papers modified with C8(1) at 80°C for 6 h

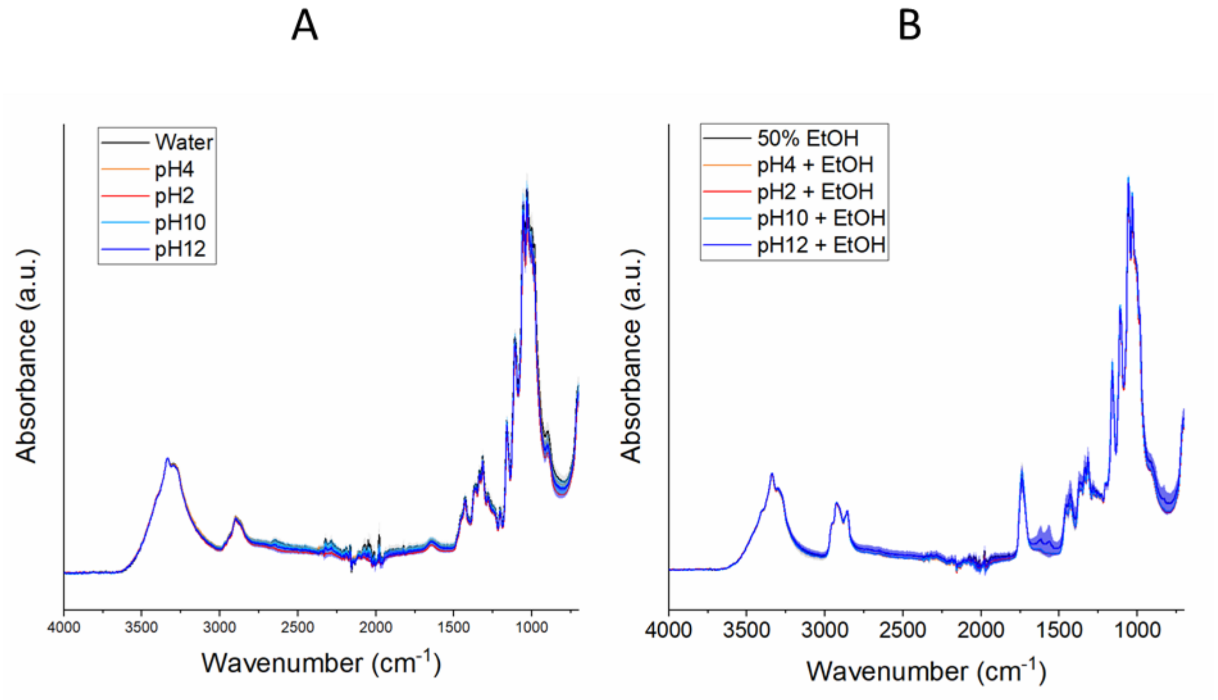

Figure S12. ATR-FTIR spectra of (A) ESC(0.8)-80T6h papers treated in water, aqueous acidic solutions of pH4 and 2, alkaline solutions of pH10 and 12, and (B) C8(1)-80T6h papers treated in 50% EtOH aqueous solution, aqueous acidic solutions of pH 4 and 2, alkaline solutions of pH10 and 12 mixed in a 1:1 ratio for one hour.

Table S5. Water contact angle (WCA) and CWC critical wicking concentration (CWC) values of covalently modified papers for various reagents, starting papers and different reaction conditions

N	Paper	Reagent	equiv.	t (h)	T (°C)	WCA	CWC
6	Gr1	C4	1	6	80	135.9 ± 2.8	27.3 ± 0.4
3	Gr1	C6	1	6	80	144.2 ± 1.3	41.3 ± 2.1
6	Gr1	C8	1	6	80	146.9 ± 0.3	46.9 ± 1.1
6	Gr1	C12	1	6	80	151.2 ± 2.1	52.8 ± 0.9
1	Gr1	C16	1	6	80	152.8 ± 0.6	54
3	Gr1	ESC	1	6	80	103.1 ± 5.1	2.7 ± 0.9
3	Gr1	POAC	1	6	80	137.4 ± 6.3	7.0 ± 2.0
3	Gr1	PAC	1	6	80	141.4 ± 1.8	9.7 ± 3.7
1	Gr1	C4	1	16	80	142.5 ± 2.5	27.5
1	Gr1	C4	1	24	80	142.3 ± 0.9	26
1	Gr1	C12	1	16	80	152.5 ± 0.9	58
1	Gr1	C12	1	24	80	152.8 ± 1.4	59.5
1	Gr1	C8	2	1	80	122.9 ± 1.1	41
1	Gr1	C8	2	2	80	130.1 ± 2.6	42
1	Gr1	C8	2	4	80	133.6 ± 0.9	45
1	Gr1	C8	2	6	80	146.1 ± 3.5	46
1	Gr1	C12	2	1	80	148.3 ± 0.2	55
1	Gr1	C12	2	2	80	148.3 ± 3.8	57
1	Gr1	C12	2	4	80	150.2 ± 0.3	56.5
1	Gr1	C12	2	6	80	150.2 ± 0.2	57
1	Gr1	C16	2	1	80	140.6 ± 2.1	48
2	Gr1	C16	2	2	80	148.9 ± 3.0	49.5 ± 0.5
1	Gr1	C16	2	4	80	151.0 ± 0.8	55
3	Gr1	C16	2	6	80	157.9 ± 3.4	57.7 ± 2.1
3	Gr1	C4	1	6	RT	127.4 ± 4.0	22.0 ± 0.1
3	Gr1	C4	1	6	40	130.3 ± 4.3	24.5 ± 0.8
3	Gr1	C8	1	6	RT	136.0 ± 5.5	46.0 ± 1.3
3	Gr1	C8	1	6	40	143.4 ± 1.8	44.0 ± 2.2

N	Paper	Reagent	equiv.	t (h)	T (°C)	WCA	CWC
3	Gr1	C12	1	6	RT	146.3 ± 3.8	46.5 ± 0.8
3	Gr1	C12	1	6	40	148.1 ± 3.6	44.5 ± 1.5
2	Gr3	C8	1	6	80	136.9 ± 2.2	35.0 ± 3.7
1	Gr17	C4	2	6	RT	129.6 ± 2.3	27.5
1	Gr17	ESC	3	6	RT	127.6 ± 1.6	16.0
1	Gr17	C12	2	6	RT	142.9 ± 4.2	46.0
1	Gr1	C16	0.8	6	80	154.0 ± 2.0	50
1	Gr1	C16	0.7	6	80	153.0 ± 2.2	49
1	Gr1	C16	0.6	6	80	146.0 ± 3.0	41
1	Gr1	C16	0.5	6	80	148.0 ± 1.9	41
1	Gr1	C12	0.6	6	80	142.3 ± 2.4	42
1	Gr1	C12	0.5	6	80	141.4 ± 2.0	27
4	Gr1	C8	0.8	6	80	145.3 ± 2.4	39.6 ± 0.2
2	Gr1	C8	0.7	6	80	147.7 ± 2.8	33.5 ± 0.5
1	Gr1	C8	0.6	6	80	149.1 ± 3.0	32
2	Gr1	C6	0.8	6	80	145.1 ± 2.0	40.0 ± 0.0
1	Gr1	C6	0.6	6	80	147.9 ± 7.1	36
2	Gr1	C4	1.5	6	80	142.7 ± 1.7	38.0 ± 0.0
3	Gr1	C4	1.2	6	80	140.6 ± 3.0	30.7 ± 0.5
3	Gr1	C4	0.8	6	80	136.9 ± 1.7	19.8 ± 3.3
1	Gr1	C4	0.6	6	80	138.8 ± 3.4	22.5
2	Gr1	POAC	1.5	6	80	147.6 ± 1.5	24.5 ± 0.5
2	Gr1	POAC	1.2	6	80	140.0 ± 4.0	22.5 ± 0.5
3	Gr1	PAC	1.5	6	80	145.4 ± 3.5	14.3 ± 0.5
3	Gr1	PAC	1.2	6	80	146.0 ± 2.6	11.5 ± 0.5
2	Gr1	ESC	1.5	6	80	137.5 ± 1.9	11.0 ± 1.0
4	Gr1	ESC	1.2	6	80	124.5 ± 4.1	9.5 ± 0.5

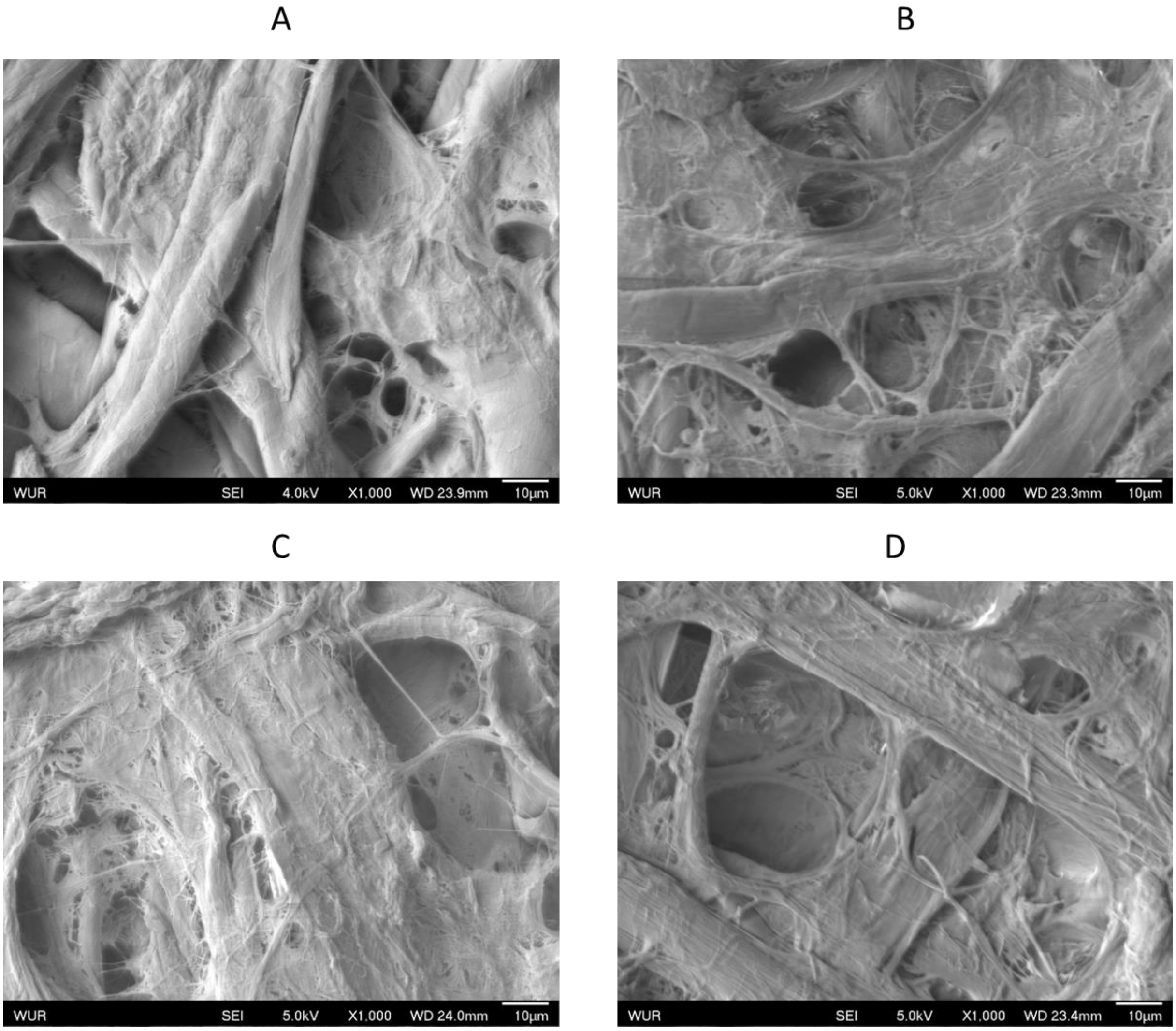

Figure S13. Scanning Electron Microscope (SEM) images of (A) bare grade 1 paper, (B) DMF-treated paper at RT, (C) DMF-treated paper at 80°C and (D) C16(0.6)-80T6h modified paper

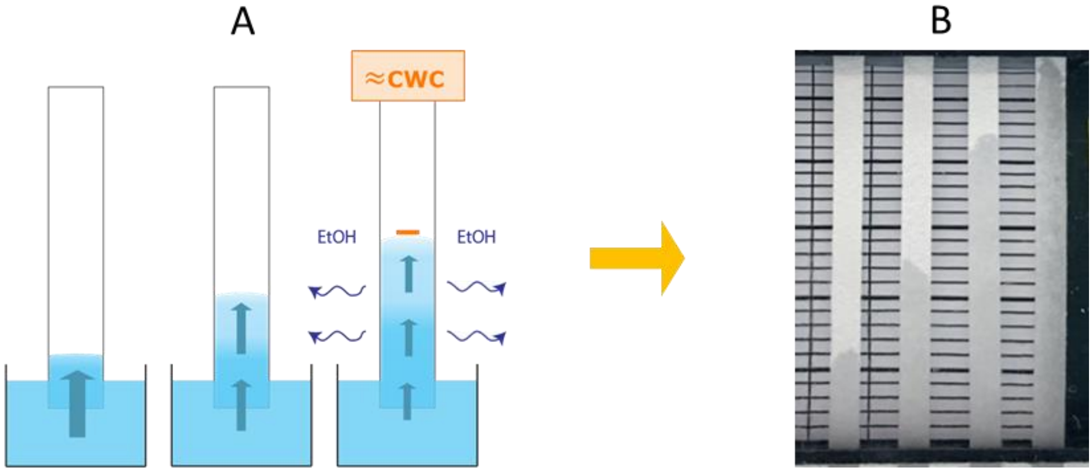

Figure S14. (A) Proposed explanation for the occurrence of a maximum flow distance in modified papers. The hypothesis to explain such a maximum flow distance is based on the evaporation of EtOH from the paper surface during the wicking process. Given that ethanol (boiling point: 78°C) is more volatile than water, flow in an open porous system will lead to evaporation at a higher rate than the evaporation of water from the binary solutions used, effectively diluting the ethanol solution. When the % EtOH is diluted to a value at or below the CWC of the modified paper, further wicking is prevented (B) An example of increasing maximum flow distances with increasing % EtOH, which were observed for C8(0.8)-80T6h paper with CWC of 42% by using 50, 60, 70 and 80% EtOH, from left to right

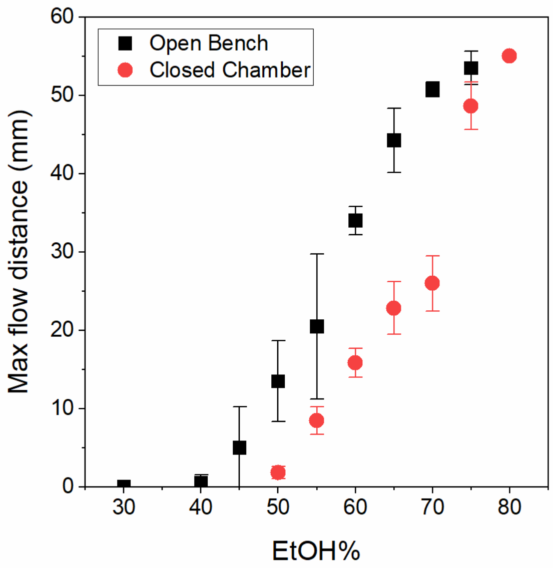

Figure S15. Maximum flow distance measurement of Gr 1 papers modified with C8(1)-80T6h on an open bench and in an air saturated closed chamber to investigate the repeatability of the measurements

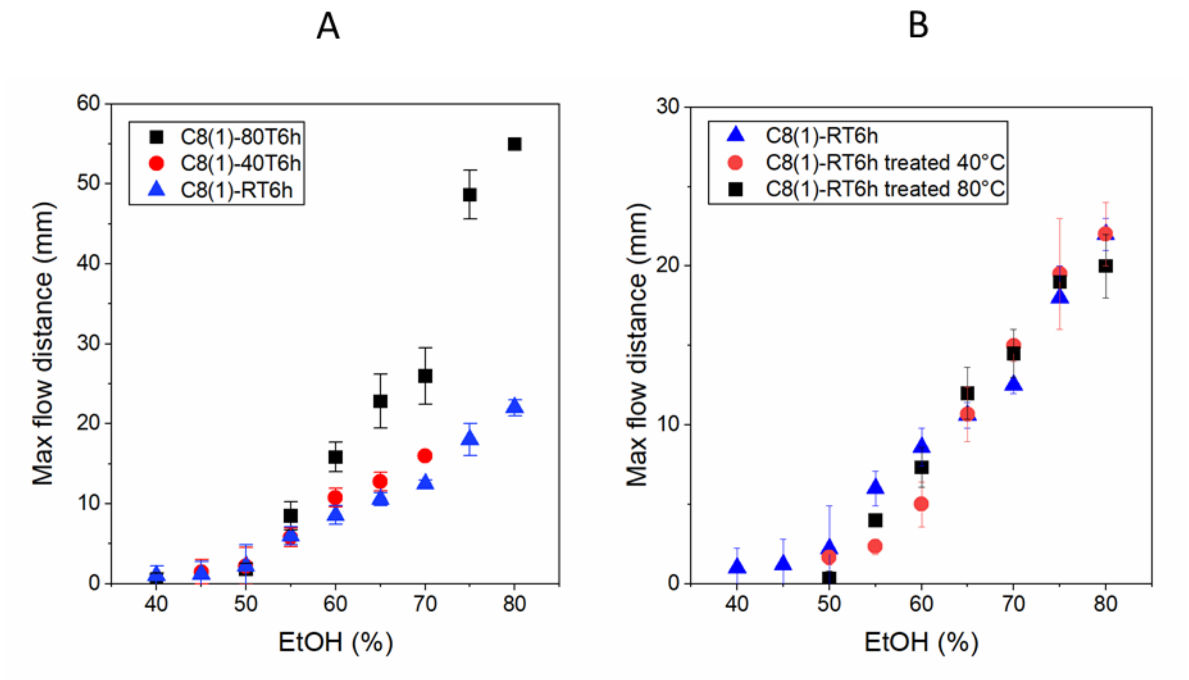

Figure S16. (A) The effect of reaction temperature on maximum flow distance for Gr1 papers modified with C8(1) paper modified at different temperatures: RT, 40ºC and 80ºC; (B) the effect of treating Gr1 C8(1)-RT6h papers in DMF at 40ºC and 80ºC for 6 h after modification

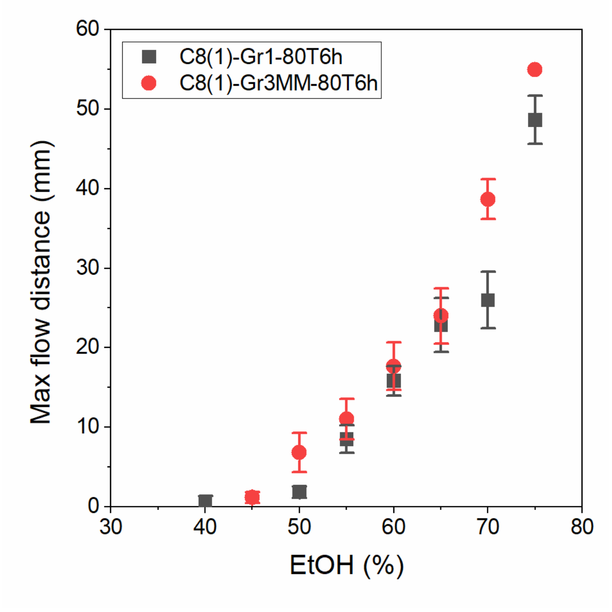

Figure S17. Maximum flow distances of C8(1)-80T6h Gr 1 and Gr 3MMpapers

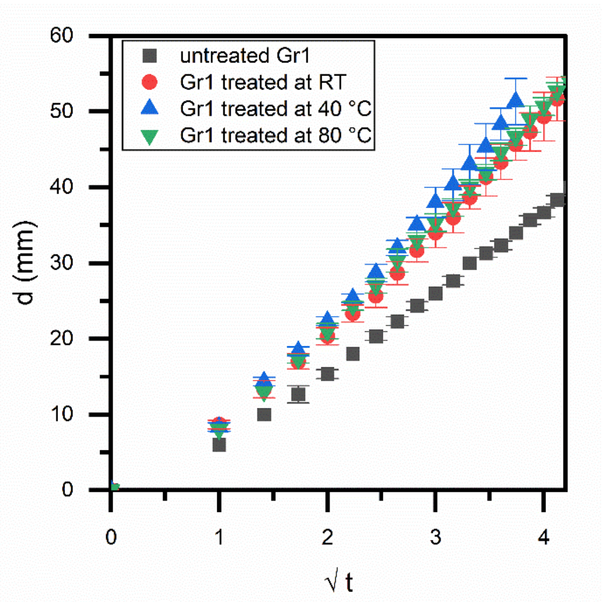

Figure S18. Distance travelled as a function of square root of time of 50% EtOH in water in untreated Gr 1 paper, and the treated papers in DMF at RT, 40ºC and 80ºC for 6 h

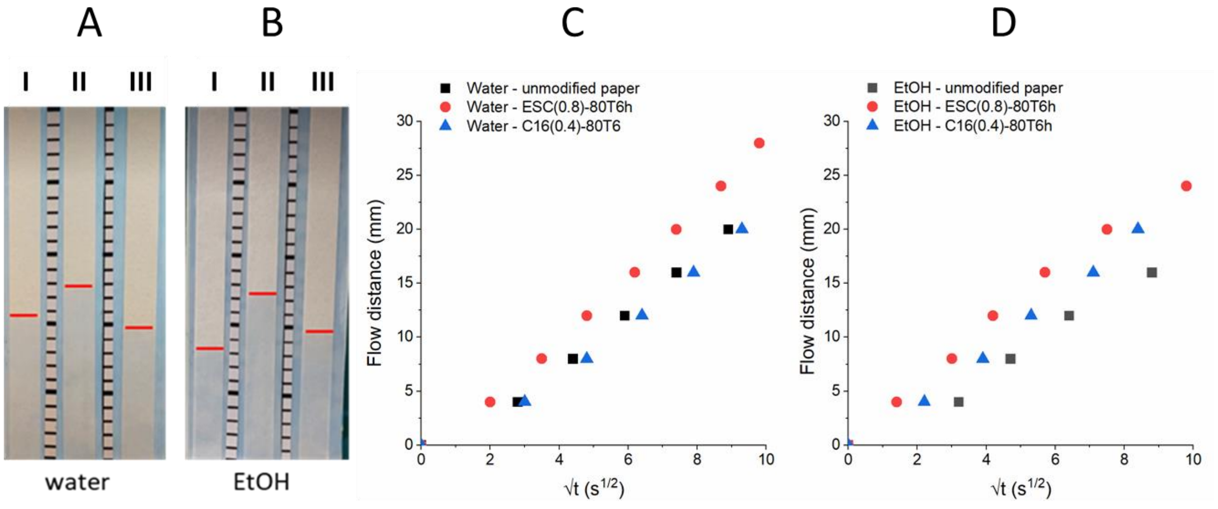

Figure S19. Varying flow rates of (A) water and (B) EtOH in (I) bare paper and hydrophobically modified yet water-permeable papers, namely (II) ESC(0.8)-80T6h and (III) C16(0.4)-80T6h, where the flow distances represent how far the liquids traveled after 80 seconds. Distance travelled as a function of square root of time are given in (C) and (D) for water and EtOH flows, respectively.

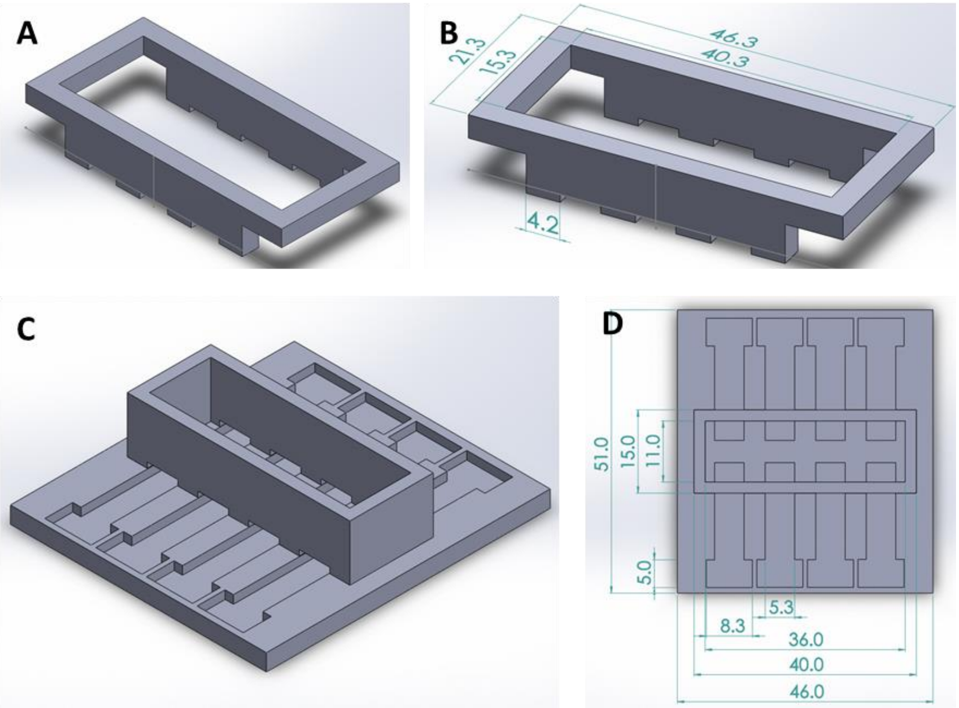

Figure S20. SolidWorks drawings of 3D printed permeability-based sensing device with dimensions (in mm): (A) isometric and (B) dimetric views of the additional part, which ensured the good contact of the sample solution and the measuring papers; (C) isometric and (D) top views of the main device.

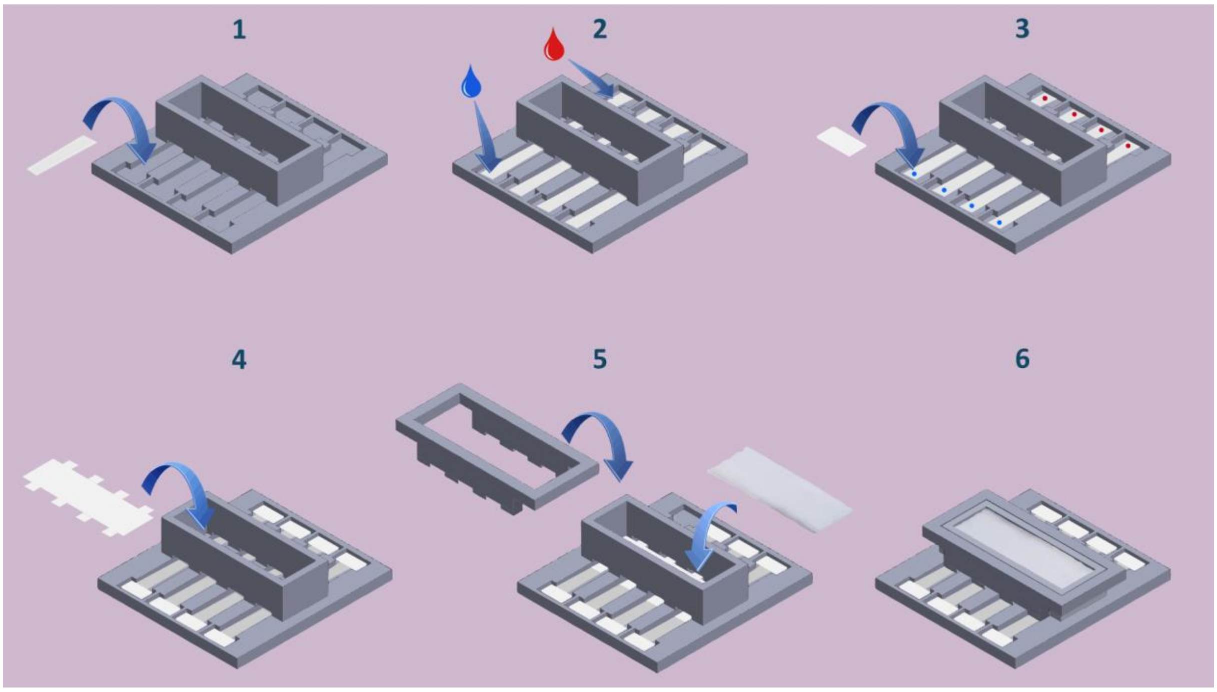

Figure S21. Assembly of the permeability-based sensing device: (1) placing hydrophobically modified papers to each channels as measuring tools with different CWC values; (2) preloading of blue (one part of device, EtOH concentrations of 10-25%) and red (the other part of device, EtOH concentration of 40-55%) food dye solution droplets to the outer edge of the paper strips to create colorimetric detection points; (3) covering those colored spots with a piece of unmodified paper; only when the modified paper with dye would be wetted - if the sample EtOH content exceeded the threshold (CWC) - would the dye be transported onto the top paper, and the color become visible; (4) placing a piece of paper sample pad, which enables the homogeneous distribution of the sample to the measuring papers from the sample reservoir into the flow channels; (5) placing a 3D-printed cover for a good contact of sample pad and the measuring paper for liquid transfer, and filling the reservoir with a cotton pad: and (6) the final assembled permeability-based sensing device.

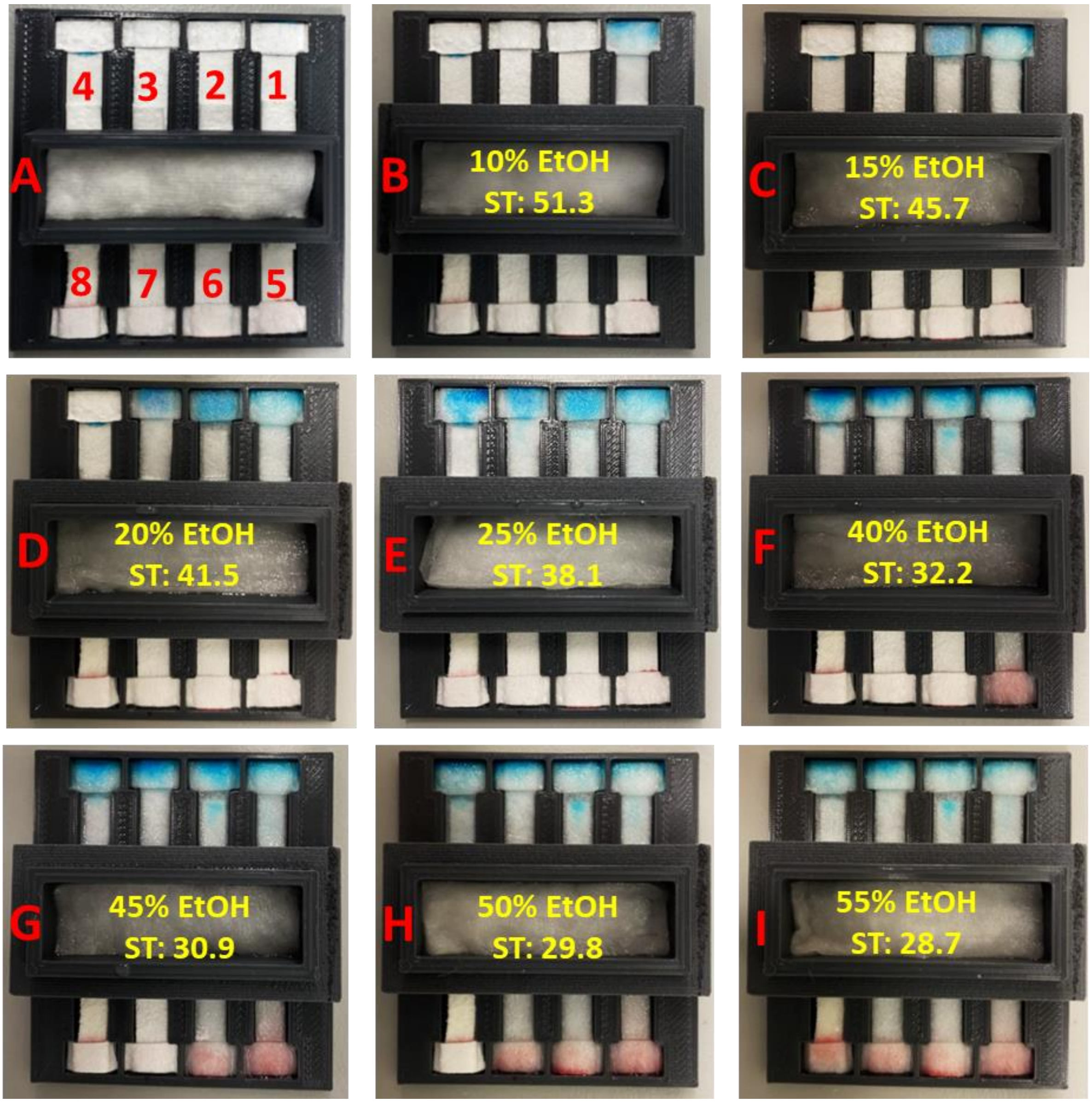

Figure S22. (A) Permeability based surface tension measurement device. Modified papers in the channels have CWC of 6, 12, 17, 22, 36, 42, 49 and 52 % EtOH corresponding to positions 1 through 8, respectively. Applied aqueous EtOH solution concentrations: (B) 10%, (C) 15%, (D) 20%, (E) 25%, (F) 40%, (G) 45%, (H) 50%, and (I) 55%, where ST stands for surface tension at 20 ℃ (mN.m-1) [2]. Note papers used from position 1 to 8: PAC(1), PAC(1.2), C4(0.8), POAC(1.2), C6(0.6), C6(1), C16(0.7), and C12(1) modified at 80 ℃ for 6 h.

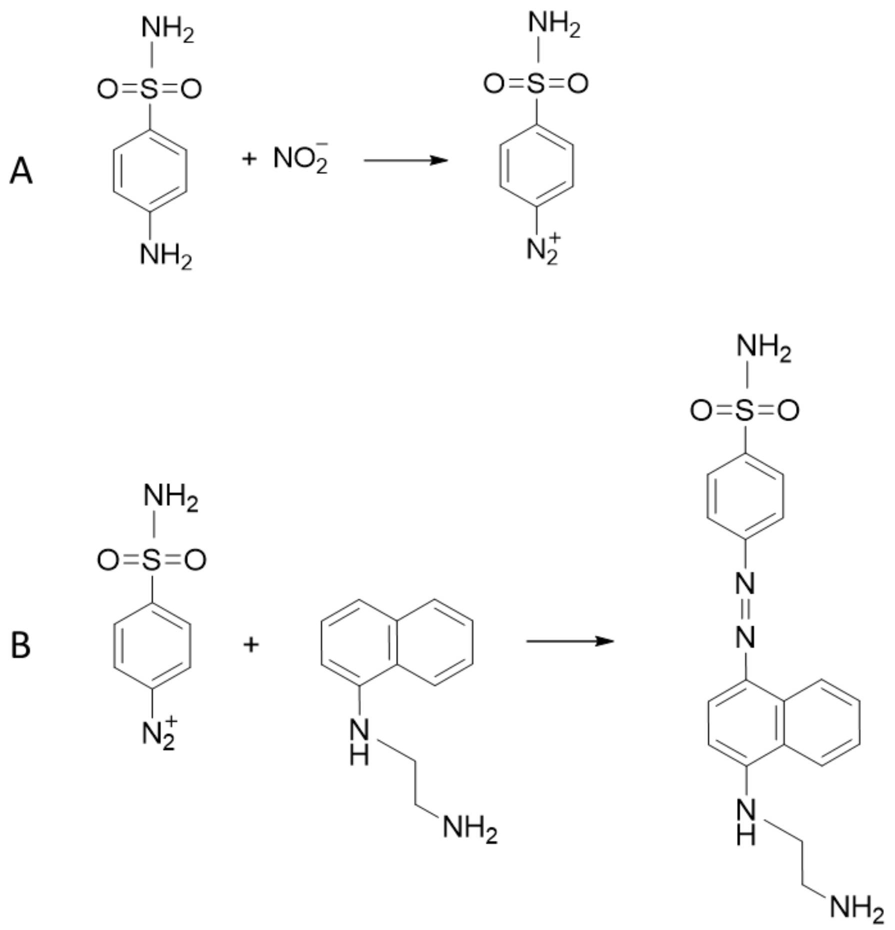

Figure S23. Multistep colorimetric Griess reaction: (A) the 1st step where nitrite reacts with sulfanilamide (SA) to produce diazonium salt intermediate product and (B) the 2nd step of Griess reaction where diazonium salt reacts with naphtyletylenediamide (NED) to produce the azo-compound that gives a purple color.[3]

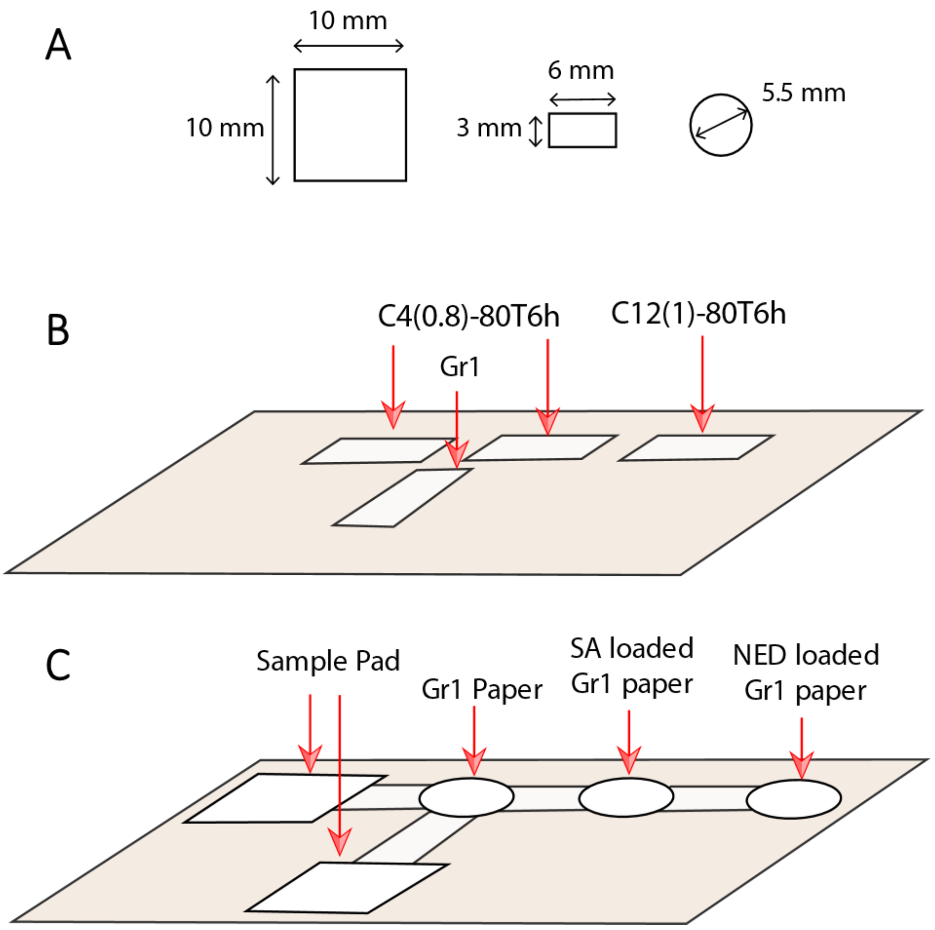

Figure S24. Assembly of permeability-based multistep valving device on a piece of adhesive backing card for nitrite detection: (A) dimensions of the papers used in the device; square sample pads (left), rectangular flow or valving papers (middle), and circular (unmodified) Gr1 papers as reaction chamber (right), (B) placing rectangular papers with the CWC of 20 and 52% EtOH (C4(0.8)-80T6h and C12(1)-80T6h, respectively) for controlled liquid flow where these papers would be used as valves, and (C) placing the circular Gr1 papers to entrap the sample solution, where 2 µL of 2% SA and 3% NED solutions are preloaded to the corresponding Gr1 papers, and the square sample pads to apply liquid sample and the ethanol

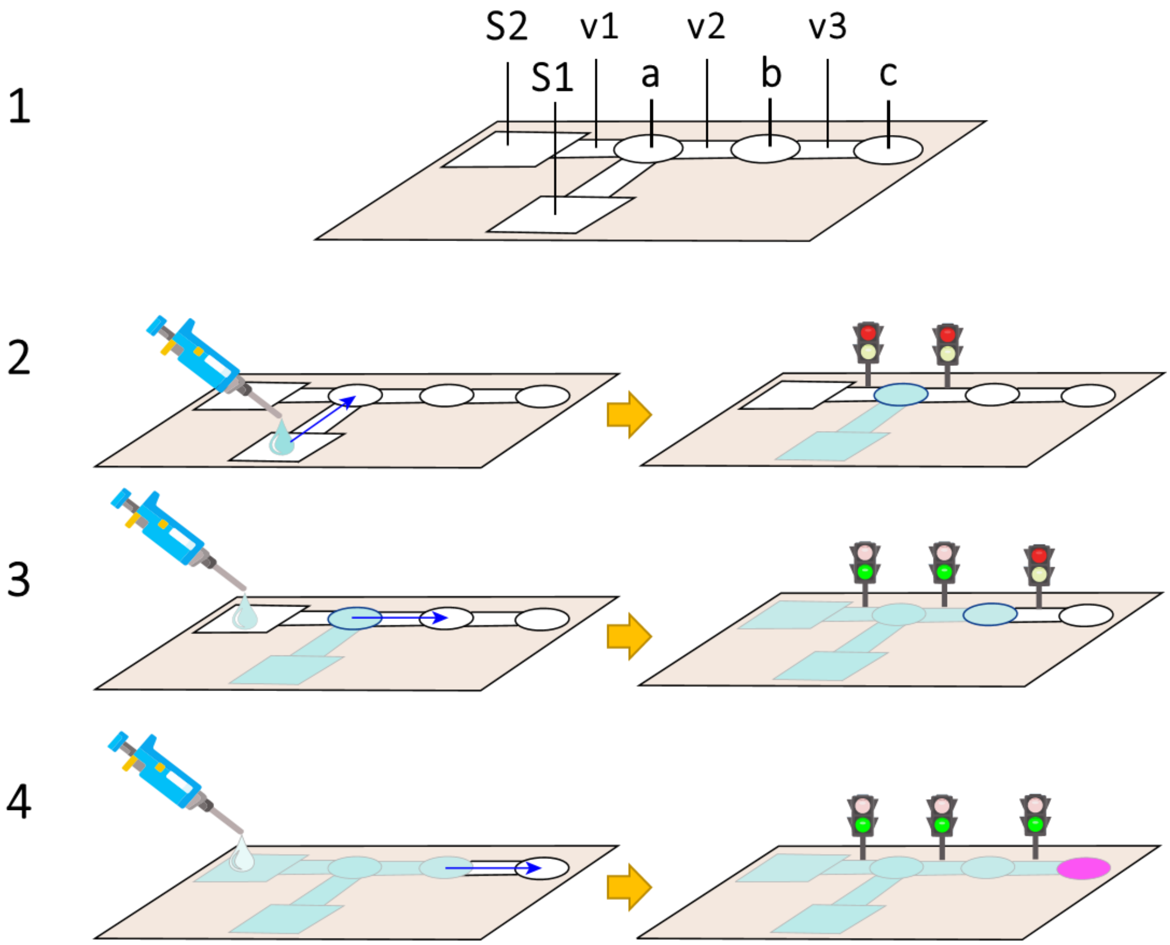

Figure S25. Schematic flow of multistep valving for nitrite determination: (1) permeability-based multistep valving device labeling; (2) loading of 50 µL of an aqueous nitrite solution to the nitrite sample pad (S1), which reaches the first circular bare paper (a) through Gr1 paper (unmodified), where flow stopped, as valve 1 (v1) and 2 (v2) would not wick water due to their CWC of 20% EtOH, thus these valves were "off"; (3) loading of 50 µL of ethanol to the ethanol pad (S2) so that the nitrite entrapped in paper (a) would reach to and be entrapped in the SA preloaded paper (b) for the first step of the Griess reaction for a minute, as v1 and v2 would be 'on' since the surface tension of the aqueous solution would decrease and the solution would wick into these valves, while the last valve (v3) still remained "off" as it had a higher CWC, 52% EtOH, than v1 and v2; (4) loading of another 50 µL of ethanol to the ethanol pad (S2) so that the diazonium salt, produced as intermediate product, in the SA paper would be transferred to the NED preloaded paper (c) to obtain the purple color response as colorimetric indication of the presence of nitrite in the sample, as v3 would be 'on' due to further decrease in the surface tension of the aqueous solution to allow wicking into this valve.

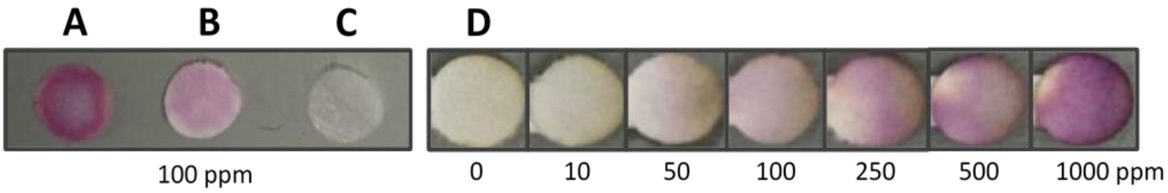

Figure S26. Color response of different reaction sequences: (A) known multistep colorimetric Griess reaction where NO_2^- aqueous solution was dropped on SA pre-loaded paper, then NED was added, (B) NO_2^- aqueous solution was dropped on SA and NED pre-loaded paper, and (C) NO_2^- aqueous solution was dropped on NED pre-loaded paper, then SA was added. (D) Color response of various concentrations of NO_2^- aqueous solution obtained from the multistep colorimetric Griess reaction (Figure S20) using the designed nitrite detection µPAD device (Figure S21 and S22)

References

[1] E. Ojogbo, R. Blanchard, and T. Mekonnen, “Hydrophobic and Melt Processable Starch-Laurate Esters: Synthesis, Structure–Property Correlations,” *J Polym Sci A Polym Chem*, vol. 56, no. 23, pp. 2611–2622, 2018, doi: 10.1002/pola.29237.

[2] G. Vázquez, E. Alvarez, and J. M. Navaza, “Surface Tension of Alcohol + Water from 20 to 50 °C,” 1995. *Journal of Chemical & Engineering Data, 40*(3), 611–614. doi: 10.1021/je00019a016

[3] L. Váradi, M. Breedon, F. F. Chen, A. Trinchi, I. S. Cole, and G. Wei, “Evaluation of novel Griess-reagent candidates for nitrite sensing in aqueous media identified via molecular fingerprint searching,” *RSC Adv*, vol. 9, no. 7, pp. 3994–4000, 2019, doi: 10.1039/c8ra07656a.
